# Structural basis for human DPP4 receptor recognition by a pangolin MERS-like coronavirus

**DOI:** 10.1371/journal.ppat.1012695

**Published:** 2024-11-08

**Authors:** Mo Yang, Zehou Li, Jing Chen, Yang Li, Ran Xu, Meihua Wang, Ying Xu, Rong Chen, Weiwei Ji, Xiaoxia Li, Jiayu Wei, Zhengrong Zhou, Minjie Ren, Ke Ma, Jiayu Guan, Guoxiang Mo, Peng Zhou, Bo Shu, Jingjing Guo, Yuan Yuan, Zheng-Li Shi, Shuijun Zhang

**Affiliations:** 1 College of Life Sciences, Nanjing Agricultural University, Nanjing, China; 2 Key Laboratory of Virology and Biosafety, Wuhan Institute of Virology, Chinese Academy of Sciences, Wuhan, China; 3 Centre in Artificial Intelligence Driven Drug Discovery, Faculty of Applied Sciences, Macao Polytechnic University, Macao, China; 4 Institute of Veterinary Medicine, Jiangsu Academy of Agricultural Sciences, Key Laboratory of Veterinary Biological Engineering and Technology, Ministry of Agriculture, Nanjing, China; 5 Guangzhou Laboratory, Guangzhou International Bio Island, Guangzhou, Guangdong, China; 6 School of Life Sciences, Anhui University, Hefei, Anhui, China; Institut Pasteur, FRANCE

## Abstract

Middle East respiratory syndrome coronavirus (MERS-CoV) and the pangolin MERS-like coronavirus MjHKU4r-CoV-1 employ dipeptidyl peptidase 4 (DPP4) as an entry receptor. MjHKU4r-CoV-1 could infect transgenic mice expressing human DPP4. To understand the mechanism of MjHKU4r-CoV-1 entry into cells, we determined the crystal structures of the receptor binding domain (RBD) of MjHKU4r-CoV-1 spike protein bound to human DPP4 (hDPP4) and Malayan pangolin DPP4 (MjDPP4), respectively. The overall hDPP4-binding mode of MjHKU4r-CoV-1 RBD is similar to that of MERS-CoV RBD. MjHKU4r-CoV-1 RBD shows higher binding affinity to hDPP4 compared to the bat MERS-like coronavirus Ty-BatCoV-HKU4. Via swapping residues between MjHKU4r-CoV-1 RBD and Ty-BatCoV-HKU4 RBD, we identified critical determinants on MjHKU4r-CoV-1 that are responsible for virus usage of hDPP4. Our study suggests that MjHKU4r-CoV-1 is more adapted to the human receptor compared to the bat HKU4 coronavirus and highlights the potential of virus emergence into the human population.

## Author summary

MjHKU4r-CoV-1 is a MERS-like coronavirus isolated in Malayan pangolins. As a pangolin coronavirus that evolves from its counterparts in bats, MjHKU4r-CoV-1 could use bat, pangolin or human dipeptidyl peptidase 4 (DPP4) as an entry receptor. MjHKU4r-CoV-1 is infectious in human organs and human DPP4 transgenic mice as well and therefore shows the risk of infecting humans. However, the mechanism of receptor recognition by MjHKU4r-CoV-1 is not clear, which is important for evaluating the zoonotic potential of the virus. Here, we showed that MjHKU4r-CoV-1 entered human DPP4 expressing cells more efficiently than its bat progenitor, Ty-BatCoV-HKU4. We further determined the crystal structures of MjHKU4r-CoV-1 receptor binding domains (RBDs) complexed with human DPP4 and pangolin DPP4, respectively. Based on structure guided mutagenesis studies, we identified key residues on MjHKU4r-CoV-1 that determined the virus preference for human DPP4 receptor. Therefore, our study implies that the pangolin coronavirus MjHKU4r-CoV-1 have a risk of emergence into the human population. Future surveillance studies of pangolins and other animals are necessary to determine the transmission chains and zoonotic potential of MjHKU4r-CoV-1.

## Introduction

Coronaviruses are positive strand RNA viruses that have been categorized into four genera, *Alpha-*, *Beta-*, *Gamma-* and *Deltacoronavirus* [1]. Three highly pathogenic betacoronaviruses, severe acute respiratory syndrome coronavirus (SARS-CoV), Middle East respiratory syndrome coronavirus (MERS-CoV) and SARS-CoV-2 have caused large outbreaks in the past twenty years [2–4]. MERS-CoV was first identified in Saudi Arabia in 2012 and since then spread globally [3,5]. As of October 2023, MERS-CoV has caused 2,608 cases of infection, resulting in 938 deaths, with a case fatality rate of 36% [6]. Although dromedary camels are potential intermediate hosts for MERS-CoV [7,8], the virus has been presumed to be originated in bats, as a large variety of MERS-like coronaviruses have been identified in different bat species [1,9–13]. Ty-BatCoV-HKU4 was isolated in lesser bamboo bats (*Tylonycteris pachypus*) and shared 77% nucleic acid sequence identity with MERS-CoV [12,13]. Both Ty-BatCoV-HKU4 and MERS-CoV were grouped within lineage C (or subgenus *Merbecovirus*) of the *Betacoronavirus* genus [12,13]. Like MERS-CoV, Ty-BatCoV-HKU4 could employ human dipeptidyl peptidase 4 (DPP4) as an entry receptor [13,14]. Human DPP4 (hDPP4) transgenic mice are also susceptible to Ty-BatCoV-HKU4 infection [13]. This suggests the risk of cross-species transmission of Ty-BatCoV-HKU4. In addition to bats, hedgehogs and pangolins have also been reported to harbour merbecoviruses [15–17]. Therefore, wild animals other than bats might also contribute to the evolution and emergence of MERS-CoV.

Coronaviruses initiate infection via binding of the viral spike (S) protein to host cell receptors [18]. Merbecoviruses have been reported to use either DPP4 or angiotensin converting enzyme 2 (ACE2) as an entry receptor [13–16,19,20]. MERS-CoV and the HKU4 coronaviruses identified in bats and pangolins, including Ty-BatCoV-HKU4, MjHKU4r-CoV-1 and pangolin-CoV-HKU4-P251T all engage DPP4 to enter cells while NeoCoV, a bat merbecovirus, use ACE2 as a functional receptor [13–16,19]. Receptor usage is a key determinant of host spectrum and therefore constitutes the species barrier for coronaviruses transmission [1,18]. For instance, the S protein of SARS-CoV-2 binds to ACE2 receptors derived from human, horseshoe bats and other species [4], which is consistent with the broad host range of the virus [21]. MERS-CoV could employ both human and dromedary camel DPP4 as entry receptors [19,22,23]. Porcine deltacoronavirus (PDCoV), a potential zoonotic swine coronavirus isolated in children with febrile illness recently [24,25], could utilize human and porcine aminopeptidase N (APN) to enter cells [26,27]. The spike proteins of coronaviruses consist of two subunits, S1 and S2 [28]. S1 is responsible for receptor binding whereas S2 subsequently mediates virus membrane fusion with host cells [28]. The receptor binding domain (RBD) is usually located on the carboxyl terminal domain (CTD) of S1 and directly engages the virus receptor expressed on the host cell surface [18,20,29–33].

We previously reported the identification and isolation of a HKU4 coronavirus in Malayan pangolins (*Manis javanica*), which is termed MjHKU4r-CoV-1[15]. MjHKU4r-CoV-1 infected human airways and intestinal organs. Moreover, it caused disease in hDPP4 transgenic mice [15]. Consistently, MjHKU4r-CoV-1 could utilize hDPP4, Malayan pangolin DPP4 (MjDPP4) and lesser bamboo bat DPP4 (TpDPP4) as entry receptors [15]. In this study, we solved the crystal structures of MjHKU4r-CoV-1 RBD in complex with human and pangolin DPP4 receptors, respectively. Based on structure guided mutagenesis studies, we identified critical determinants on MjHKU4r-CoV-1 that accounts for its preference to hDPP4 compared to the bat HKU4. Our study indicates that MjHKU4r-CoV-1 engages human and pangolin receptors via a conserved binding mode, supporting the idea that pangolins may serve as important hosts for merbecoviruses. Therefore, pangolin coronaviruses pose a risk of crossing species barrier and may potentially result in emergence into the human population.

## Results

### Complex structures of MjHKU4r-CoV-1 RBD bound to human and pangolin DPP4

**Fig 1:**
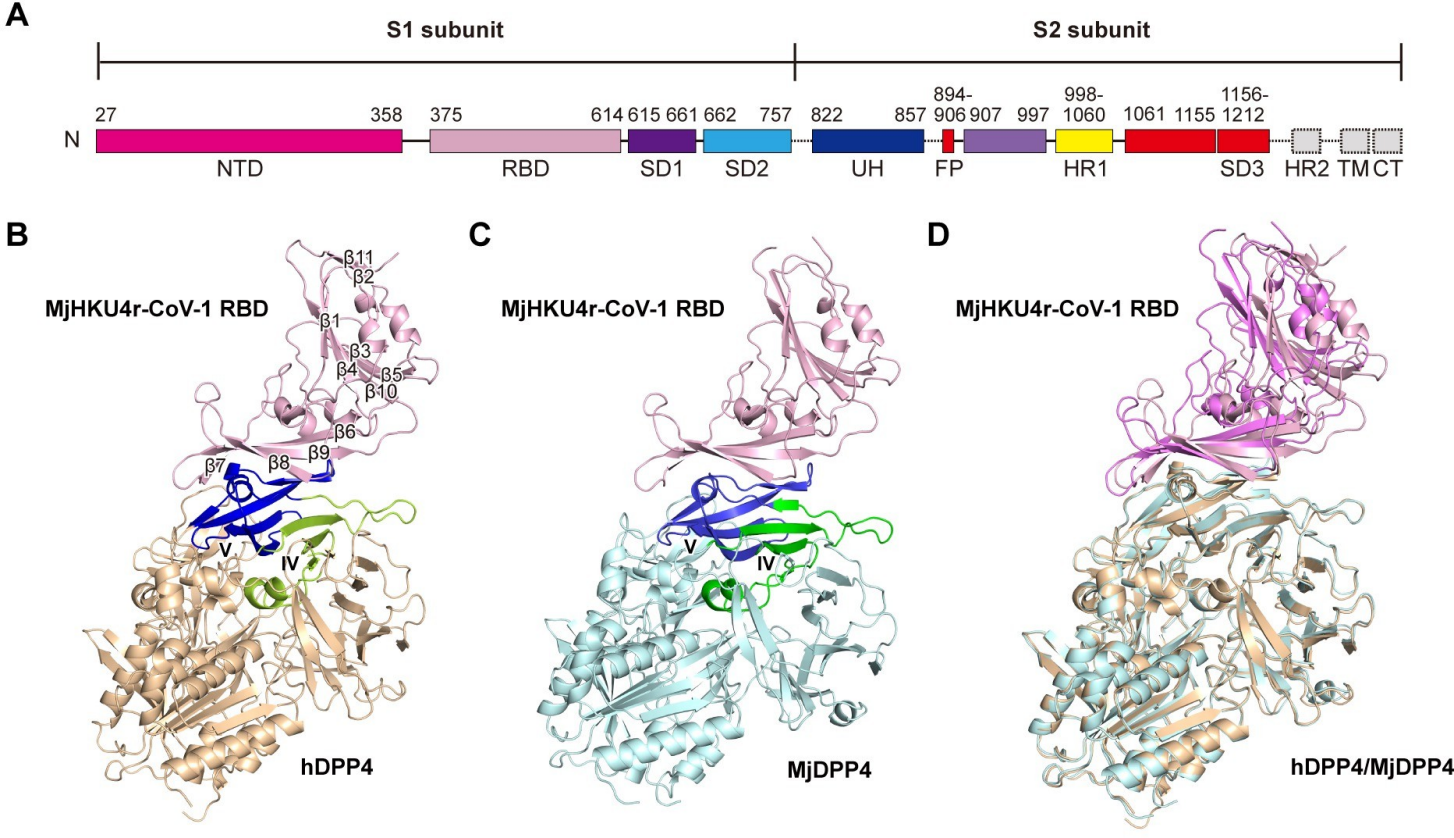
Overall structure of MjHKU4r-CoV-1 RBD in complex with human DPP4 (hDPP4) or pangolin DPP4 (MjDPP4). **(A)** Schematic diagram of the MjHKU4r-CoV-1 spike (S) protein ectodomain. NTD, N-terminal domain of S1. RBD, receptor binding domain. SD, subdomain. UH, upstream helix. FP, fusion peptide. HR, heptad repeat. TM, transmembrane domain. CT, cytoplasmic tail. **(B)** Crystal structure of MjHKU4r-CoV-1 RBD bound to hDPP4. MjHKU4r-CoV-1 RBD and hDPP4 are colored in light pink and wheat, respectively. Blades IV and V of hDPP4 are highlighted in lemon and blue, respectively. MjHKU4r-CoV-1 RBD adopts a β barrel structure and contains eleven β-strands. **(C)** Crystal structure of MjHKU4r-CoV-1 RBD bound to MjDPP4. MjHKU4r-CoV-1 RBD and MjDPP4 are colored in light pink and pale cyan, respectively. Blades IV and V are highlighted in green and blue. **(D)** Superposition of MjHKU4r-CoV-1 RBD-hDPP4 and MjHKU4r-CoV-1 RBD-MjDPP4 complexes. The two structures exhibit high similarity and the RMSD between them is 0.468 Å.

We previously showed that MjHKU4r-CoV-1 bound to human, bat and pangolin DPP4 [15]. Similar to MERS-CoV and Ty-BatCoV-HKU4, MjHKU4r-CoV-1 RBD is located on the CTD of the S1 subunit [15] (Fig 1A). To characterize the mechanism of cross-species receptor recognition by MjHKU4r-CoV-1, we solved the crystal structures of the viral RBD complexed with hDPP4 and MjDPP4 to resolutions of 2.6 Å and 2.7 Å, respectively (Fig 1B and 1C and S1 Table), which allows for detailed analysis of the interaction between the pangolin coronavirus and its two host receptors. Specifically, MjHKU4r-CoV-1 RBD (residues 375–614), hDPP4 (residues 39–766) and MjDPP4 (residues 39–766) were all expressed in Hi5 insect cells, purified by Ni-NTA affinity purification and size exclusion (S1A–S1C Fig). The two complexes were formed via co-crystallization of the viral RBD and hDPP4 / MjDPP4. There are two RBD/DPP4 heterocomplexes related by the non-crystallographic 2-fold axes in the asymmetric units (ASU) of both complex structures (S2A and S2B Fig). The two MjHKU4r-CoV-1 RBD-MjDPP4 in the ASU are almost identical, with a RMSD of 0.335 Å over 936 aligned Cα atoms (S2C Fig). The two MjHKU4r-CoV-1 RBD-hDPP4 heterocomplexes in the ASU are also similar to each other (RMSD of 0.787 Å over 944 aligned Cα atoms, S2D Fig). MjHKU4r-CoV-1 RBD is composed of a core structure and an external domain. The core structure folds as a five-stranded β sheet with short helices sitting on top. As the core structure is in the distal end far away from the RBD-receptor interface, it does not directly contact DPP4 (Fig 1B and 1C). The external domain, or the receptor binding motif (RBM), consists of a four-stranded (β6-β9) antiparallel β sheet. The lateral side of the RBM directly engages the DPP4 receptors (Fig 1B and 1C). The overall structure of MjHKU4r-CoV-1 RBD is similar to those of other merbecoviruses, including MERS-CoV, Ty-BatCoV-HKU4, Pi-BatCoV-HKU5 and NeoCoV [14,20,34,35], with root mean square deviation (RMSD) values ranging from 0.8 Å to 1.2 Å for corresponding aligned 208, 198 and 187 Cα atoms, respectively (S3A Fig). However, the conformation of the β6-β7 loop, the β8 strand and the β8-β9 loop in MjHKU4r-CoV-1 RBM displays obvious differences to those of other merbecoviruses (S3A Fig). The β6-β7 loop of MjHKU4r-CoV-1 RBD was longer than that of NeoCoV and therefore would clash with the α10 helix of ACE2 (S3B Fig). Reciprocally, the β8 strand of NeoCoV RBM was tilted downwards by 23.6° compared to that of MjHKU4r-CoV-1 RBM (S3A Fig). As a result, NeoCoV RBD would collide with blade V of DPP4 (S3C Fig). This would explain why NeoCoV does not use DPP4 but instead employs ACE2 as a functional receptor [20]. Therefore, the large structural differences in the RBMs between MjHKU4r-CoV-1 and NeoCoV would account for their discrepancies in receptor usage. This reveals that coronaviruses could evolve to use different receptors via only minor changes in the RBMs, adding to the complexity of receptor usage. The structure of hDPP4 or MjDPP4 consists of a α/β hydrolase domain and an eight-bladed β propeller domain. Both DPP4 receptors bind to MjHKU4r-CoV-1 RBM via blades IV and V of their β propeller domains (Fig 1B and 1C), in agreement with the complex structures of MERS-CoV RBD-hDPP4 and Ty-BatCoV-HKU4 RBD-hDPP4 solved previously [14,36]. As MjDPP4 shares 89.3% amino acid sequence identity with hDPP4, the structures of these two DPP4 molecules highly resemble each other, with a RMSD of 0.435 Å over 728 aligned Cα pairs (S3D Fig). However, when hDPP4 and MjDPP4 are superimposed, there is a 4.8° tilting angle between the respective MjHKU4r-CoV-1 RBDs bound to them (Figs 1D and S4A). The orientation of MjHKU4r-CoV-1 RBD with respect to DPP4s also differs from those found for other RBD-DPP4 complexes (S4B–S4D Fig). Although MjHKU4r-CoV-1 RBD and Ty-BatCoV-HKU4 RBD share high homology in sequence (78% sequence identity) and structure (RMSD of 0.8 Å over 208 aligned Cα atoms), their centers are separated by 9.6° when MjDPP4 and hDPP4 of respective complexes are aligned (S4B Fig), which would result in slight difference of respective viral RBD footprints on receptors. Similarly, for the SARS-related coronaviruses, including SARS-CoV, SARS-CoV-2 Omicron variant and the bat coronavirus RaTG13, the orientations their RBDs with respect to the ACE2 receptors could differ up to 7° (S4E and S4F Fig). The divergence of approach angles of these viral RBDs are probably contributed by the inherent flexibilities between the core structures and the RBMs as well.

### MjHKU4r-CoV-1 binds to structurally conserved regions on human and pangolin DPP4

**Fig 2:**
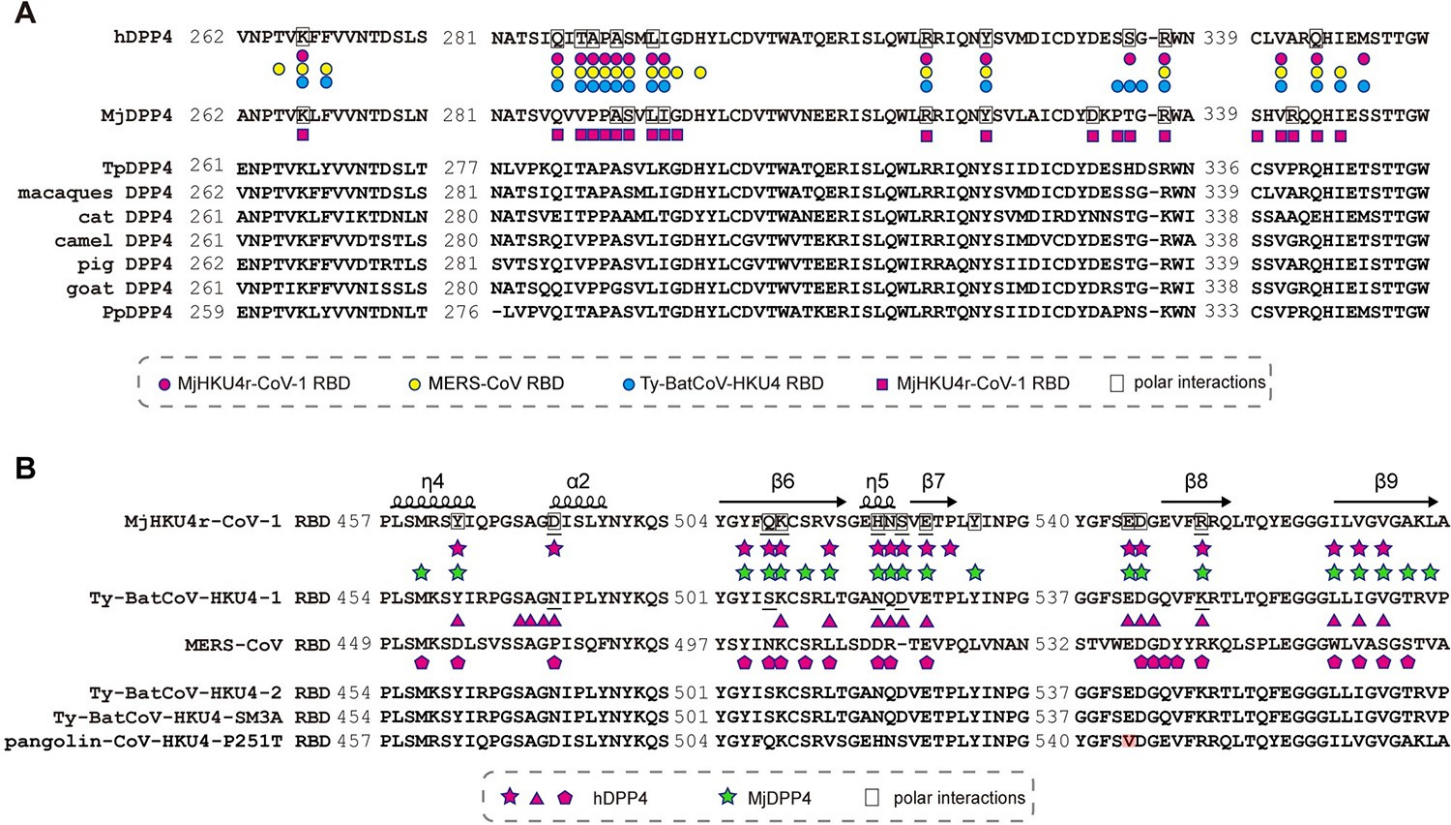
Structure based sequence alignment of different DPP4s and merbecoviruses RBDs. **(A)** Sequence alignment of hDPP4 (NP_001926.2), MjDPP4 (XM_017664375.2), TpDPP4 (MH345671.1), macaques DPP4 (NM_001039190.2), cat DPP4 (NM_001009838.1), camel DPP4 (XP_006176870.1), pig DPP4 (NM_214257.1), goat DPP4 (KF574265.1) and PpDPP4 (AGF80256.1). Residues on hDPP4 or MjDPP4 that bind to MjHKU4r-CoV-1 RBD, MERS-CoV RBD, and Ty-BatCoV-HKU4 RBD are labelled according to the code of the key below the sequences. **(B)** Sequence alignment of MjHKU4r-CoV-1 RBD (UVJ46720.1), Ty-BatCoV-HKU4-1 RBD (ABN10848.1), MERS-CoV RBD (JX869059), Ty-BatCoV-HKU4-2 RBD (EF065506.1), Ty-BatCoV-HKU4-SM3A RBD (MW218395.1) and pangolin-HKU4-P251T RBD (OM009282.1). Compared to MjHKU4r-CoV-1 RBM, the non-conserved V544 residue in pangolin-CoV-HKU4-P251T RBM is highlighted in pink. The secondary structure elements are generated with ESPript1. Residues on viral RBDs that bind to hDPP4 are labelled according to the code of the key below the sequences. Genbank accession numbers of corresponding sequences in (A) and (B) are enclosed in brackets.

The surface areas buried at the interfaces of MjHKU4r-CoV-1 RBD-hDPP4 complex and MjHKU4r-CoV-1 RBD-MjDPP4 complex are 1,524.9 Å^2^ (783.1 Å^2^ on hDPP4 and 741.8 Å^2^ on RBD) and 1,754.8 Å^2^ (925.1 Å^2^ on MjDPP4 and 829.7 Å^2^ on RBD), respectively, which are comparable to those of MERS-CoV RBD-hDPP4 complex (1,036.6 Å^2^ on hDPP4 and 925.9 Å^2^ on RBD) or Ty-BatCoV-HKU4 RBD-hDPP4 complex (893.7 Å^2^ on hDPP4 and 798.5 Å^2^ on RBD), but much larger than that of NeoCoV RBD-batACE2 complex (480.3 Å^2^ on bat ACE2 and 411.4 Å^2^ on RBD). Therefore, the different approach angle of MjHKU4r-CoV-1 RBD with respect to MjDPP4 leads to a 15% increase in the BSA. Residues located on MjHKU4r-CoV-1 RBD-hDPP4 or MjHKU4r-CoV-1 RBD-MjDPP4 interface are identified using a distance cutoff of 4.0 Å. A total of 16 hDPP4 and 21 MjDPP4 residues are in contact with MjHKU4r-CoV-1 RBD, respectively. 12 out of the 16 RBD contacting residues on hDPP4 are identical to their counterparts on MjDPP4 (Fig 2A). Reciprocally, there are 17 and 20 residues on MjHKU4r-CoV-1 RBD that contact hDPP4 and MjDPP4, respectively, with 15 RBD residues shared by the two DPP4 molecules (Fig 2B). The larger BSA between MjHKUr-CoV-1 RBD and MjDPP4 also results in slight changes of the contact residues on the binding interface. Compared to the MjHKUr-CoV-1 RBD-hDPP4 complex, there are extra contacts between MjHKUr-CoV-1 RBD and MjDPP4, which involve residues M460, S511, Y525, A567 and L569 of viral RBD, and G296, D331, P333, S339 and R342 of MjDPP4 (Fig 2A and 2B and S2 and S3 Tables). However, the overall binding modes of MjHKU4r-CoV-1 RBD to hDPP4 and MjDPP4 are similar, suggesting MjHKU4r-CoV-1 recognizes pangolin and human receptors in a conserved manner.

**Fig 3:**
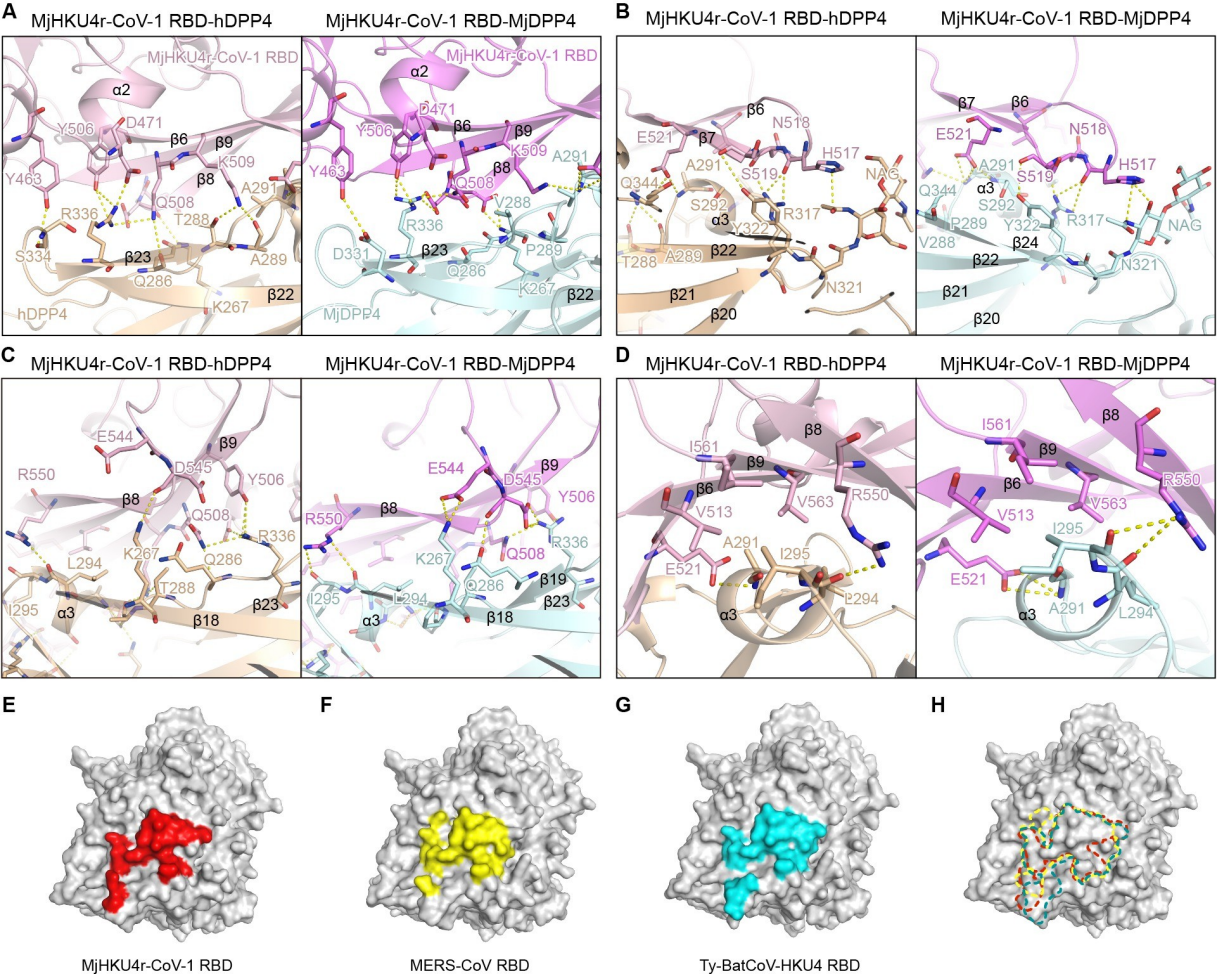
MjHKU4r-CoV-1 recognizes conserved residues on human and pangolin DPP4 receptors. (A-D) Atomic details of the interaction between MjHKU4r-CoV-1 RBD and hDPP4/MjDPP4. MjHKU4r-CoV-1 RBD, hDPP4 and MjDPP4 are colored in light pink, wheat and pale cyan, respectively. Contacting residues on respective proteins are represented as sticks, with nitrogen and oxygen atoms colored in blue and red, respectively. MjHKU4r-CoV-1 RBD and hDPP4/MjDPP4 are colored as in Fig 1B and 1C. (E-G) Footprints of MjHKU4r-CoV-1 RBD (E), MERS-CoV RBD (F) and Ty-BatCoV-HKU4 RBD (G) on hDPP4. hDPP4 is represented as gray surface. Residues on hDPP4 contacting these three RBDs are colored in red, yellow and cyan, respectively. (H) Overlay of MjHKU4r-CoV-1 RBD, MERS-CoV RBD and Ty-BatCoV-HKU4 RBD footprints on hDPP4. The boundaries of these RBD footprints are circled with red, yellow and blue dotted lines, respectively.

Extensive networks of conserved electrostatic interactions and hydrogen bonds are distributed across the interfaces of these two complexes, which involved residues on the η4 helix, the α2 helix and the β6-β9 strands of MjHKU4r-CoV-1 RBD, and their interacting partners on the blade IV and V of hDPP4 and MjDPP4 (S2 Table). Specifically, the RBD residue Y463 interacts with S334 of hDPP4 or D331 of MjDPP4 (Figs 3A and S5A). Y506 and Q508 are hydrogen bonded to R336 of hDPP4 or MjDPP4 (Figs 3A and S5A). K509 forms hydrogen bond interactions with T288 and A289 of hDPP4, or A291 in MjDPP4 (Figs 3A and S5A and S2 and S3 Tables). The equivalents of K509 on MERS-CoV and Ty-BatCoV-HKU4, which are K502 and K506, have also been shown to be involved in binding to hDPP4 in previous studies [14,34]. The backbone carbonyl oxygen atoms of H517 and N518 in MjHKU4r-CoV-1 RBD interact with the side chain of R317 in hDPP4 or MjDPP4 via hydrogen bonding (Figs 3B and S5B). S519 is hydrogen bonded to Y322 of hDPP4 or MjDPP4 (Figs 3B and S5B). E521 is in contact with A291 and Q344 of hDPP4, or A291, S292 and Q344 of MjDPP4 (Figs 3B and S5B). R550 interacts with L294 and I295 of hDPP4 or MjDPP4 (Figs 3C and 3D and S5C and S5D). As MjHKU4r-CoV-1 RBD is orientated differently on hDPP4 and MjDPP4, there are also interactions specific to each complex. The RBD residue D471 only forms a salt bridge interaction with hDPP4 R336 (Figs 3A and S5A). In contrast, E544 contacts K267 of MjDPP4 via a salt bridge (Figs 3C and S5C). The corresponding residue in Ty-BatCoV-HKU4, E541, is essential for viral RBD interacting with hDPP4 [14]. In the MjHKU4r-CoV-1 RBD-hDPP4 complex, D545 forms ionic interactions with K267 of hDPP4 and therefore D545 plays a similar role as E544 in the MjHKU4r-CoV-1 RBD-MjDPP4 complex. D545 also forms an ionic bond with R336 of MjDPP4 (Figs 3C and S5C). The side chains of RBD residues V513, E521, R550, I561 and V563 pack against A291, L294 and I295 of hDPP4 or MjDPP4 (Figs 3D and S5D and S3 Table), forming a hydrophobic center across the interface. In addition, H517 is hydrogen bonded to the glycan linked to N321 of hDPP4 or MjDPP4 near one end of the interface (Figs 3B and S5B). As expected, the footprint of MjHKU4r-CoV-1 RBD on hDPP4 largely overlaps with those of MERS-CoV RBD and Ty-BatCoV-HKU4 RBD (Fig 3E–3H). The conserved receptor binding mode highly suggests that these three merbecoviruses probably evolve from the same ancestor.

The aforementioned key residues on MjHKU4r-CoV-1 RBD (D471, K509, E521, R550) were individually mutated to alanine and the affinity of each mutant RBD protein to hDPP4 or MjDPP4 was measured using surface plasmon resonance (SPR) assay (Table 1 and S6A and S6B Fig). D471A, K509A and R550A mutation decreased the affinity of MjHKU4r-CoV-1 RBD to hDPP4 or MjDPP4 by 10- to 30-fold (Table 1 and S6A and S6B Fig), while E521A mutation slightly lowered the affinity by 3- to 5-fold (Table 1 and S6A and S6B Fig). Unexpectedly, although D471 is not in direct contact with residues in MjDPP4, D471A mutation showed a 10-fold reduction in affinity of MjHKU4r-CoV-1 RBD to MjDPP4 (Table 1 and S6B Fig), which is likely contributed by allosteric effects. The side chain of D471 forms a network of hydrogen bond interactions, either directly, or through water molecules, with Y506 and Q508 of MjHKUr-CoV-1 RBD (S6C Fig), both of which contact MjDPP4. Additionally, we performed 100-ns molecular dynamics simulations for both the wild-type and mutant (D471A) MjHKU4r-CoV-1 RBD complexed with MjDPP4 to further evaluate the effects of D471A mutation. The total binding affinity of the complex decreased in the mutant systems (S4 Table), which was consistent with the affinity experiment above. Further results indicated that the D471A mutation allosterically weakened the binding of Y506 and Q508 with MjDPP4. The D471A mutation reduced the binding free energy contribution of Y506 from -1.61 kcal/mol to -0.76 kcal/mol (S6D Fig). Meanwhile, the D471A mutation also reduced the number of contacts involving Y506 and Q508, possibly allosterically affecting their interactions with MjDPP4 (S6E Fig).

**Table 1:** The affinities of wild-type (WT) or mutant MjHKU4r-CoV-1 RBD/Ty-BatCoV-HKU4 RBD and hDPP4/MjDPP4 measured by SPR.

	hDPP4	MjDPP4
MjHKU4r-CoV-1 RBD	0.28±0.07 μM	0.17±0.06 μM
MjHKU4r-CoV-1 RBD E521A	1.23±0.05 μM	0.62±0.08 μM
MjHKU4r-CoV-1 RBD K509A	9.17±1.42 μM	2.61±1.37 μM
MjHKU4r-CoV-1 RBD R550A	2.42±0.85 μM	1.41±0.08 μM
MjHKU4r-CoV-1 RBD D471A	8.45±3.51 μM	2.67±0.79 μM
MjHKU4r-CoV-1 RBD R550K	2.02±0.38 μM	
MjHKU4r-CoV-1 RBD D471N	3.59±1.00 μM	
MjHKU4r-CoV-1 RBD H517N	50.43±48.64 μM	
MjHKU4r-CoV-1 RBD S519D	3.03±0.38 μM	
MjHKU4r-CoV-1 RBD Q508S	3.07±0.78 μM	
Ty-BatCoV-HKU4 RBD	5.22±1.98 μM	
Ty-BatCoV-HKU4 RBD K547R	0.51±0.21 μM	
Ty-BatCoV-HKU4 RBD N468D	1.59±0.27 μM	
Ty-BatCoV-HKU4 RBD N514H	3.67±2.73 μM	
Ty-BatCoV-HKU4 RBD D516S	1.07±0.07 μM	
Ty-BatCoV-HKU4 RBD S505Q	2.78±0.34 μM	

These mutagenesis studies confirmed that disruption of the interaction across the MjHKU4r-CoV-1 RBD-DPP4 interface substantially affected binding of respective RBD and receptor. D471A, K509A and R550A mutation on MjHKU4r-CoV-1 RBD also decreased its affinity to TpDPP4 by about 6-fold, respectively (S6F Fig), suggesting MjHKU4r-CoV-1 may bind to similar regions on the bat DPP4 receptor.

### Determinants of human receptor usage of pangolin HKU4 coronaviruses

**Fig 4:**
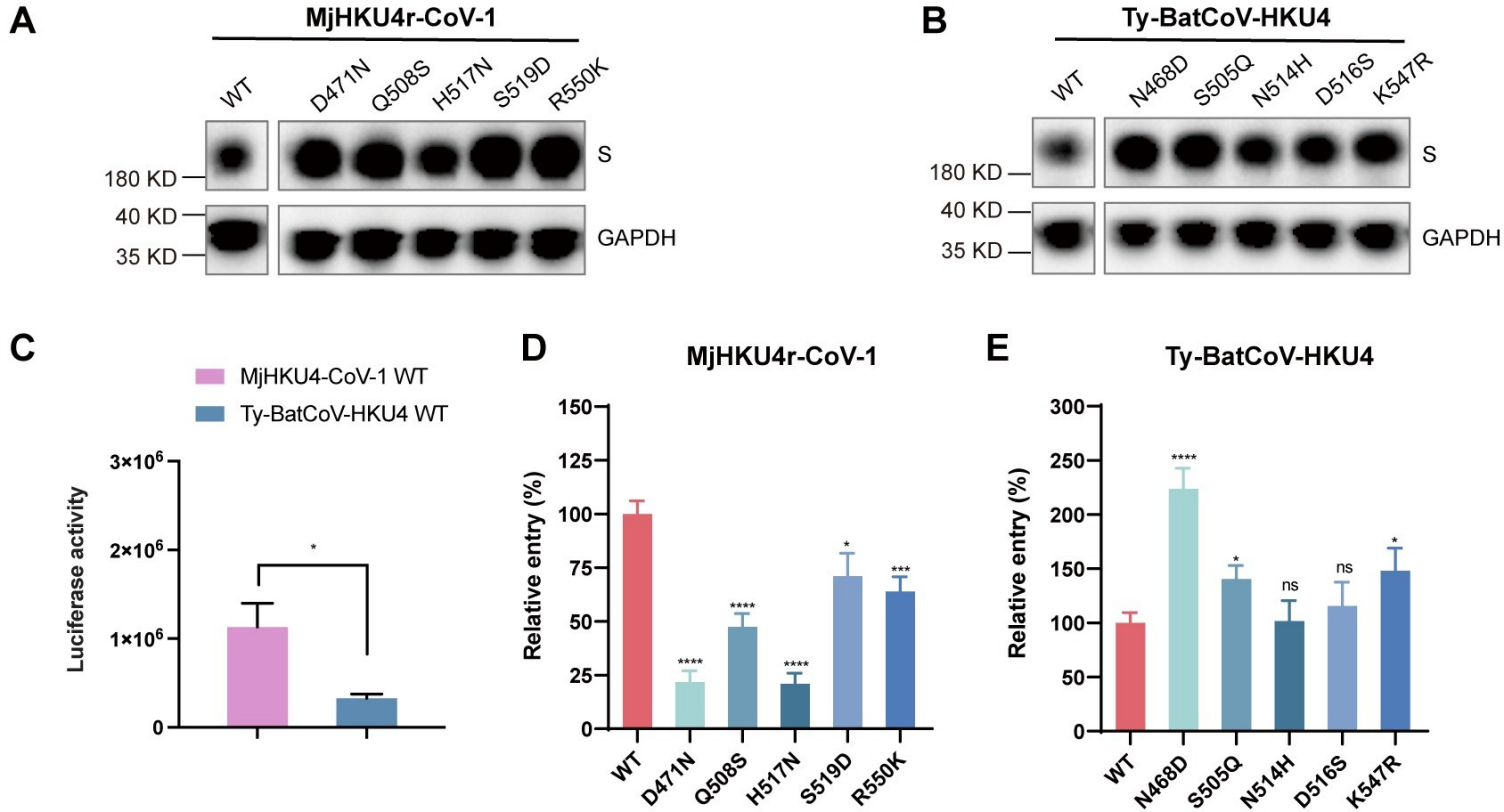
Effect of residue mutation on MjHKU4r-CoV-1 and Ty-BatCoV-HKU4 entry into cells. **(A, B)** Expression of WT or mutant MjHKU4r-CoV-1 (A) and Ty-BatCoV-HKU4 (B) spike proteins was detected in HEK293T cells. HEK293T cells were transfected with WT or mutant spike protein expression plasmid containing a C-terminal S-tag. At 48 h post transfection (h.p.t.), the cells were harvested, lysed, and subjected to western blotting analysis using the anti-S-tag antibody. Expression of full-length spike proteins and GAPDH was shown. **(C)** Comparison of the entry efficiencies between wild-typed (WT) MjHKU4r-CoV-1 and Ty-BatCoV-HKU4 spike protein packaged pseudoviruses in HEK293T-hDPP4 cells. Luciferase activity was determined at 48 h.p.t.. **(D, E)** Entry efficiency of pseudotyped viruses bearing the WT or mutant MjHKU4r-CoV-1 (D) and Ty-BatCoV-HKU4 (E) spike proteins in HEK293T-hDPP4 cells was determined by measuring luciferase activity at 48 h.p.t.. Data are presented as means and standard errors of the means (SEMs) of triplicate assays. Statistical significance was assessed using a two-tailed Student’s *t*-test. *P* < 0.05 was considered statistically significant. *P < 0.05; **P < 0.01; ***P < 0.001; ****P < 0.0001; ns, no significant difference.

Three bat-derived HKU4 coronaviruses, Ty-BatCoV-HKU4-1, Ty-BatCoV-HKU4-2 and Ty-BatCoV-HKU4-SM3A, also use hDPP4 as an entry receptor [13–15]. Residues on Ty-BatCoV-HKU4-1 RBD that are in contact in hDPP4 are identical to the corresponding residues in the latter two viruses (Fig 2B). However, the hDPP4 contacting residues are not strictly conserved between Ty-BatCoV-HKU4-1 RBD and MjHKU4r-CoV-1 RBD (Fig 2B). We therefore compared the differences in receptor usage between bat and pangolin derived coronaviruses. The SPR assay showed that the affinity of MjHKU4r-CoV-1 RBD to hDPP4 was about 20-fold higher than that of Ty-BatCoV-HKU4 RBD to hDPP4 (Table 1, S6A and S7A Figs), implying the former virus was probably more adapted to human DPP4 receptor. To further characterize the determinants of hDPP4 usage, we first compared the footprint of MjHKU4r-CoV-1 RBD on hDPP4 with that of Ty-BatCoV-HKU4 (Fig 2B). Among the 17 residues on MjHKU4r-CoV-1 RBD that contact hDPP4, 6 are identical to corresponding residues on Ty-BatCoV-HKU4 RBD. These include K509/K506, P523/P520, E521/E518, D545/D542 of MjHKU4r-CoV-1/Ty-BatCoV-HKU4, respectively, which are also strictly conserved in MERS-CoV RBD. 5 residues are of similar biochemical properties, including V513/L510, N518/Q515, R550/K547, I561/L558 and V563/I560 of MjHKU4r-CoV-1/Ty-BatCoV-HKU4, respectively. Residues in the remaining 4 positions are D471/N468, Q508/S505, H517/N514, S519/D516 of MjHKU4r-CoV-1/Ty-BatCoV-HKU4, respectively. Based on the above structure guided sequence alignment, we mutated 5 hDPP4 contacting residues on MjHKU4r-CoV-1 RBD to their counterparts on Ty-BatCoV-HKU4, including D471/N468, Q508/S505, H517/N514, S519/D516, R550/K547. The binding affinity of each recombinantly expressed mutant RBD protein to hDPP4 was measured using SPR, which revealed that mutation of MjHKU4r-CoV-1 RBD residues D471, Q508, H517, S519 and R550 individually decreased the binding affinity by 5- to 10-fold (S7B Fig). These single mutations already decreased the association rate constants (k_a_) of MjHKU4r-CoV-1 RBD binding to hDPP4 to the level of k_a_ of Ty-BatCoV-HKU4 RBD binding to hDPP4, while the dissociation rate constants of binding were marginally affected (S5 Table), suggesting these mutations mainly affected the rate of complex formation but not complex dissociation. R550K mutation was associated with an increase of mutation energy (ΔΔG) by 1.17 kcal/mol, which was largely contributed by the van der Waals terms [37]. This suggested R550K mutation lowered the affinity of binding mainly through decreasing van der Waals contacts between MjHKU4r-CoV-1 RBD and hDPP4. On the contrary, corresponding reciprocal mutations on Ty-BatCoV-HKU4 RBD (N468, S505, N514, D516 and K547) increased binding affinity by 2- to 10-fold (S7A Fig). The effect of above mutations on viral infectivity was further evaluated by the pseudotyped virus entry assay. HEK 293T cells stably expressing human DPP4 were infected with the pseudotyped virus carrying S protein from MjHKU4r-CoV-1 or Ty-BatCoV-HKU4. MjHKU4r-CoV-1 pesudovirus showed higher cell entry efficiency than Ty-BatCoV-HKU4 pseudovirus (Fig 4A–4C), which was consistent with the binding affinities of the two viral RBDs to hDPP4 (Table 1, S6B and S6F Fig). Mutation of D471, Q508, H517, S519 and R550 on MjHKU4r-CoV-1 RBD to the corresponding residues on Ty-BatCoV-HKU4 RBD reduced the pseudotyped virus entry in varying degrees, with D471N and H517N mutants decreased the amount of the pseudotyped virus entering cells by more than 75%, while Q508S, S519D and R550K only slightly suppressed virus entry (Fig 4A and 4D). Reciprocally, mutations on Ty-BatCoV-HKU4 S protein, including N468, S505 and K547 increased entry of Ty-BatCoV-HKU4 pseudovirus. The N468D mutation doubled the amount of virus entering cells (Fig 4B and 4E). Taken together, we have identified critical residues on HKU4 coronaviruses that potentially determine host range.

## Discussion

The cross-species transmission of coronaviruses poses huge threats to the public health. We previously reported isolation of a pangolin MERS-like coronavirus, MjHKU4r-CoV-1, which could use hDPP4 as an entry receptor and is pathogenic in hDPP4 transgenic mice [15], thus having the risk of emergence into human. In this study, we reported the crystal structures of MjHKU4r-CoV-1 RBD complexed with hDPP4 and MjDPP4, respectively. MjHKU4r-CoV-1 RBD binds to structurally conserved regions on hDPP4 and MjDPP4, which indicates that the virus engages the pangolin and human receptors via similar modes. Based on structure guided mutagenesis, we identified residues on MjHKU4r-CoV-1 RBD that account for its stronger binding to hDPP4 compared to the closely related bat coronavirus Ty-BatCoV-HKU4. Moreover, our pseudovirus infection assay revealed that swapping residues on the two viral RBDs increased the entry of MjHKU4r-CoV-1 while inhibited the entry of Ty-BatCoV-HKU4. We noticed that the effect of the RBD mutations on the pseudovirus entry was not totally consistent with the protein binding result in the SPR assay. The SPR assay measured the affinity between the viral RBD and the receptor. However, the pseudovirus infection assay was more complex and was affected by multiple factors. While the binding affinity between the RBD and the viral receptor played the most important role during the pseudovirus entry process, the incorporation of the S protein, cleavage of the S protein and the conformations of the S protein trimers might also affect the efficiency of pseudoviruses entry. In summary, we have identified key determinants of host receptor usage on MjHKU4r-CoV-1 RBD. When this paper was in preparation, another group reported the crystal structure of MjHKU4r-CoV-1 RBD bound to hDPP4 [38]. While they identified residues on DPP4 derived from multiple species that were important for RBD binding, determinants on MjHKU4r-CoV-1 RBD that accounts for its broad host range was not clarified. Coronaviruses frequently mutate to adapt to new host receptors, but the virus receptors seldom mutate. Therefore, in this study, we revealed key residues on MjHKU4r-CoV-1 that account for its differential receptor usage.

In addition to MjDPP4 and hDPP4, MjHKU4r-CoV-1 live virus could use DPP4 orthologs from multiple other mammalian species, including macaques, bats, camels, goats, pigs and cats, to enter cells [15]. Sequence alignment reveals that residues on MjDPP4 or hDPP4 that are responsible for binding to MjHKU4r-CoV-1 are mostly conserved among DPP4 molecules derived from these species (Fig 2A), which indicates that the virus also probably binds to corresponding residues on other homologous DPP4 molecules of potential animal hosts. A recent study reported isolation of another pangolin HKU4 coronavirus, pangolin-CoV-HKU4-P251T, which also caused disease in hDPP4 transgenic mice [16]. The S proteins of MjHKU4r-CoV-1 and pangolin-CoV-HKU4-P251T share 99.3% sequence identity. Similar to MjHKU4r-CoV-1, live pangolin-CoV-HKU4-P251T virus could infect hDPP4 and MjDPP4 overexpressing cells. Mapping of hDPP4 binding residues on MjHKU4r-CoV-1 to those of pangolin-CoV-HKU4-P251T reveals only one amino acid difference (Fig 2B), we therefore postulate that pangolin-CoV-HKU4-P251T likely interact with the same set of residues on hDPP4 and MjDPP4 as MjHKU4r-CoV-1. Consistent with the structural analysis, sequence comparison of the RBDs among pangolin and bat HKU4 coronaviruses show that they differ mainly in the RBM regions, with bat coronaviruses resemble each other more closely than their counterparts in pangolin coronaviruses and vice versa (Fig 2B). The non-conserved residues in the RBMs among these HKU4 coronaviruses may be attributed to the different evolutionary pathway of bat and pangolin coronaviruses. Bats are the natural reservoirs of MERS like coronaviruses. Till now, the closest relative of MERS-CoV was NeoCoV, which was found in Cape Serotine bats (*Neoromicia capensis*) [20]. Ty-BatCoV-HKU4 identified in lesser bamboo bats and pangolin MjHKU4r-CoVs belonged to the same species and their close genetic relationship suggested a possible bat-pangolin transmission of the virus species [15]. However, it is not clear whether pangolins get infected by these MERS-like coronaviruses directly from bats or from other animals [15]. Even though there have been no reports of human infection of pangolin MERS-like coronavirus yet, by comparing the receptor binding mechanisms of bat and pangolin MERS-like coronaviruses, our study indicates that the latter use human receptors more efficiently to infect cells than their bat progenitors and thus show higher zoonotic potential. Therefore, our study also necessitates surveillance of animals to determine the transmission chain and spillover risk of these coronaviruses.

The protein binding and pseudovirus infection assays revealed that swapping key residues between Ty-BatCoV-HKU4 RBD and MjHKU4r-CoV-1 RBD changed the preference of respective virus to hDPP4 (S7A and S7B Fig and Fig 4D and 4E). This was probably caused by the alteration of the interactions between respective RBD and hDPP4. For instance, D471 on MjHKU4r-CoV-1 RBD forms a strong salt bridge interaction with R336 of hDPP4 (Figs 3A and S5A and S2 Table). Mutation of D471 to the corresponding residue (N468) of Ty-BatCoV-HKU4 would probably disrupt this salt bridge while changing N468 to glutamate on Ty-BatCoV-HKU4 would introduce extra binding to hDPP4. The hydrogen bonding between Q508 of MjHKU4r-CoV-1 RBD and hDPP4 Q286 would also likely to be abolished when mutating Q508 to S505 of Ty-BatCoV-HKU4 (Figs 3A and S5A and S2 Table). H517N mutation on MjHKU4r-CoV-1 RBD significantly reduced protein binding and MjHKU4r-CoV-1 pseudovirus infection (Table 1 and S7B Fig and Fig 4D), as this mutation would likely break the hydrogen bonding between the side chain of H517 and the NAG linked to N321 of hDPP4 (Figs 3B and S5B and S2 Table). However, the corresponding N514H mutation on Ty-BatCoV-HKU4 RBD did not affect the pseudovirus infection. We infer that a single N514H may not be able to restore its interaction with the NAG of hDPP4, as the β6-β7 loop of Ty-BatCoV-HKU4 RBD on which N514 is located shows large conformational difference to that of MjHKU4r-CoV-1 RBD (S3A Fig). Based on the virus replication in cell lines derived from different species, the pangolin HKU4 coronavirus MjHKU4r-CoV-1 also shows broader host range than bat HKU4 [13,15,16]. MjHKU4r-CoV-1 could efficiently infect cells overexpressing TpDPP4 [15], which was consistent with our SPR result that detected binding of MjHKU4r-CoV-1 RBD to TpDPP4 [15]. We noticed that Zhao et al. reported no binding of these two proteins in the SPR assay [38]. The discrepancy could be caused by the difference in the status of proteins prepared, such as the oligomeric states of the recombinant RBDs being tested. The same set of residues on MjHKU4r-CoV-1 RBD would probably also determine virus binding to other homologous DPP4 receptors.

The core structures of the RBDs of DPP4 binding merbecoviruses are conserved both in sequences (~60% sequence identity) and structures (S3A and S8A Figs), while the RBMs of DPP4-binding merbecoviruses exhibit greater sequence diversity (45% sequence identity). Compared to the core structures, the receptor binding residues located on β6-β9 strands of the RBMs are mostly structurally conserved (S8B Fig). These residues are located on the central regions of the RBD-receptor interfaces and thus would be responsible for maintaining the virus-receptor interaction. However, other receptor binding residues on the helices or loops connecting the β strands display greater sequence and structural variance (S8A–S8C Fig). These residues are located on the peripheral regions of the RBD-receptor interfaces. Therefore, the DPP4-binding merbecoviruses would be able to adjust their affinities to receptors only via changing the RBM residues located on the peripheral regions of the virus-receptor interface. NeoCoV, a bat merbecovirus, has been found to display the highest genome identity (85%) to MERS-CoV so far and belong to the same species as MERS-CoV. However, the RBM of NeoCoV only shows 15% sequence identity to that of MERS-CoV (S8A Fig). While the typical four-stranded antiparallel β sheet is preserved in NeoCoV RBM, its β8 strand would collide with hDPP4 (S3C Fig). Therefore, merbecoviruses are able to fine-tune their affinities to certain receptors or even switch to different receptors only via minor structural changes on the viral RBMs while preserve the structures of the other parts during evolution.

The structure of MjHKU4r-CoV-1 complexed with hDPP4 revealed that the pangolin coronavirus directly engages human receptor in a way similar to that of other merbecoviruses, including MERS-CoV and Ty-BatCoV-HKU4, providing evidence supporting pangolins as potential important hosts for coronaviruses. This study also indicates the spillover risk of pangolin HKU4 coronaviruses. The structures solved provide valuable targets for design of pan-merbecoviruses drugs.

## Material and methods

### Cell lines and virus

Hi5 and Sf9 insect cells were maintained in the SIM HF medium and SIM SF medium (Sino Biological Inc., Beijing, China) at 27°C, respectively. HEK293T cells and HEK293T cells stably expressing human DPP4 (HEK293T-hDPP4) were maintained in Dulbecco’s modified Eagle’s medium (DMEM, Gibco) containing 10% fetal bovine serum (FBS, Gibco).

### Protein expression and purification

The human DPP4 (hDPP4, GenBank accession number: NP_001926, residues 39–766), *Manis javanica* DPP4 (MjDPP4, residues S39 to P766, NCBI Reference Sequence: XM_017664375.2), *Tylonycteris pachypus* bat DPP4 (TpDPP4, residues S38 to P763, GenBank accession number: MH345671.1), Ty-BatCoV HKU4-RBD (HKU4-RBD, residues E372-Y611, Genbank accession number: ABN10848.1), MERS-CoV-RBD (MERS-RBD, GenBank accession number: JX869059, residues E367 to Y606) and MjHKU4r-CoV-1-RBD (MjHKU4r-RBD, GenBank accession number: UVJ46720.1, residues 375–614) proteins were expressed using the Bac-to-Bac baculovirus expression system (Invitrogen). The coding sequences for MjHKU4r-RBD, MERS-RBD, HKU4-RBD, MjDPP4 and TpDPP4 ectodomains, were codon-optimized for insect cells and synthesized by the Beijing Tsingke Biotech. The coding sequence of hDPP4 ectodomain was amplified from the Huh7 cells. These genes were individually cloned into the pFastbac1 (Invitrogen) vector at the *Pst* I and *Eco*R I restriction sites. All proteins contained an N-terminal gp67 signal peptide and a C-terminal 8×His tag. Transfection and virus amplification was done in Sf9 cells, and recombinant proteins were expressed in Hi5 cells. The target proteins, secreted in the Hi5 cell culture supernatants, were purified by Ni-NTA affinity chromatography (HisTrap FF, Cytiva), followed by purification with anion exchange (Resource Q column, Cytiva) and size exclusion chromatography (Superdex 200 Increase 10/300 GL column, Cytiva).

### Protein crystallization

For protein crystallization, monomeric MjHKU4r-RBD and hDPP4/MjDPP4 were concentrated to 5 mg/mL and mixed at a 1:1 stoichiometry. The proteins were crystallized by the sitting-drop vapor diffusion method at 18°C with 1μl of protein solution mixed with 1μl of reservoir buffer. The crystals of the MjHKU4r-RBD and hDPP4 complex were obtained in 0.1 M HEPES (pH 7.0) and 10% (wt/vol) PEG 6000. For the MjHKU4r-RBD and MjDPP4 complex, crystals were obtained in 0.1 M Ammonium tartrate dibasic (pH 7.0) and 12% (wt/vol) PEG 3350.

### Data collection and structure determination

Crystals were cryoprotected in 20% (vol/vol) glycerol in reservoir solution. The diffraction data were collected at the Shanghai Synchrotron Radiation Facility beamline BL02U1. All data were indexed, integrated, and scaled with XDS [39]. The structures were solved by molecular replacement in Phaser program [40], using Ty-BatCoV-HKU4 RBD/hDPP4 (PDB code: 4QZV) as the search model. Further rounds of iterative model building and refinement were performed using phenix.refine [41] and COOT [42], respectively. The stereochemical quality of the final model was assessed with Molprobity [43]. Data collection, processing, and refinement statistics are summarized in Table S1. Buried surface areas of different complexes were calculated by the PISA program [44]. The structural figures were generated using PyMOL [45].

### Surface plasmon resonance assay

The SPR assays were carried out at room temperature (25°C) using a BIAcore T200 system. hDPP4, MjDPP4 and TpDPP4 proteins were chemically immobilized with 1-ethyl-3-(3-dimethylaminopropyl) carbodiimide (EDC) and N-hydroxysuccinimide (NHS) to the CM5 sensor chips (Cytiva). Serially diluted wild-type or mutant RBD protein dissolved in HEPES buffer (20 mM HEPES, pH 7.4, 150 mM NaCl, and 0.005% (v/v) Tween-20) were used to flow over the chip surface. After each cycle, the sensor surface was regenerated using 17 mM NaOH. The binding kinetics were analyzed with the BIAevaluation software (Version 4.1) using the 1:1 Langmuir binding model.

### Simulation systems preparation

To get an explanation at the atomic level, the initial 3D structure of MjHKU4r-CoV-1 RBD bound with MjDPP4 was extracted from the crystal structure acquired from this study (PDB code: 8ZE6). Then, *in silico* site mutation was performed utilizing PyMOL [45]. Structure optimization was performed using Schrödinger [46] to avoid atom clashes.

### Molecular dynamic simulations

Molecular dynamics simulations have been conducted for MjDPP4 in complex with the wild-type and D471A mutant of MjHKU4r-CoV-1 RBD, focusing exclusively on the RBD and MjDPP4. All systems were conducted for two 100 ns replicates using the AMBER 22 suite [47] in explicit water. The files of topology and coordinate were generated using Tleap [48] with the ff14SB AMBER force field [49]. System neutrality was maintained by adding Na^+^ and Cl^−^ ions as needed. The models were solvated with TIP3P water molecules [50] in a rectangular box. Four steps in molecular dynamics simulations were conducted including energy minimization, heating, equilibration, and production runs. At first, energy minimization was applied to each system through a multistep process, utilizing both the steepest descent and conjugate gradient algorithms. Next, the systems were incrementally heated from 0 K to 300 K over 10,000 steps. Following this, under the conditions of 300 K and 1.0 atm, a 200-ps equilibration was performed for all systems. During the three steps mentioned above, the heavy atoms are limited with a force constant of 5.0 kcal/mol/Å^2^. Finally, unrestrained MD production simulations were carried out.

### Trajectory analysis

Detailed analyses were conducted for the last 10 ns trajectories. The binding affinity between the RBD and MjDPP4 was evaluated by using the conventional Molecular Mechanics Generalized Born Surface Area (MM/GBSA) approach [51] in AMBER tools. To identify the difference of key residues contributing to complex binding, binding free energy decomposition was performed using the MM/GBSA approach. Additionally, contact analysis between the RBD and MjDPP4 was further conducted using VMD [52], which involved residues located at the binding surface.

### Pseudovirus production and transduction

Pseudovirus particles bearing wild-type and mutant S proteins of MjHKU4r-CoV-1 and Ty-BatCoV-HKU4 were generated in HEK293T cells. Briefly, wild-type and mutant MjHKU4r-CoV-1 and Ty-BatCoV-HKU4 S-expressing plasmids were transfected into HEK293T cells, respectively. At 24 h post transfection, the cells were transduced with VSV-ΔG-Fluc and incubated for 2 h. Then the inoculum was removed, and the cells were washed with DMEM and maintained in fresh medium. At 48 h post transduction, the supernatant containing pseudovirus particles was harvested by removing cell debris. Pseudoviruses were quantified by qRT-PCR as previously described and normalized to the same amount before transduction [15]. 293T-hDPP4 cells were transduced with pseudoviruses. Entry efficiency was measured at 48 h post transduction by determining luciferase activity using a Bright-Lite Luciferase Assay System (Vazyme) and a microplate reader (BioTek).

## Supporting information

S1 Fig **The size exclusion chromatograms of MjHKU4r-CoV-1 RBD (A), hDPP4 (B) and MjDPP4 (C).** The proteins were purified by a Superdex 200 Increase 10/300 GL column (Cytiva). The pooled proteins, which are indicated by arrows, were further analyzed by SDS-PAGE. Lane 1, protein molecular weight marker. Lane 2, pooled proteins.
(TIF)

S2 Fig Comparisons of the heterocomplexes in the asymmetric units (ASU) of the structures of two RBD/DPP4 complexes. The two MjHKU4r-CoV-1 RBD-hDPP4 **(A)** and two MjHKU4r-CoV-1 RBD-MjDPP4 **(B)** heterocomplexes in the ASUs are related by non-crystallographic 2-fold axes (represented as lens-shaped symbols). **(C)** Structural alignments of the two MjHKU4r-CoV-1 RBD-MjDPP4 complexes in the ASU. **(D)** Superimposition of two MjHKU4r-CoV-1 RBD-hDPP4 complexes in the ASU.
(TIF)

S3 Fig Structural comparison of merbecoviruses RBD and receptors. **(A)** Superimposition of the structures of MjHKU4r-CoV-1 RBD (PDB code: 8ZDX), Ty-BatCoV-HKU4 RBD (PDB code: 4QZV), MERS-CoV RBD (PDB code: 4KR0), Pi-BatCoV-HKU5-CoV RBD (PDB code: 5XGR) and NeoCoV RBD (PDB code: 7WPO). Zoom-in view of RBM from different merbecoviruses was shown. **(B)** Alignment of MjHKU4r-CoV-1 RBD to NeoCoV RBD-ACE2 complex (PDB code: 7WPO). The arrow indicates clashes between MjHKU4r-CoV-1 RBD and ACE2. **(C)** Alignment of NeoCoV RBD to MjHKU4r-CoV-1 RBD-hDPP4 complex. The arrow points to clashes between NeoCoV RBD and hDPP4. **(D)** Comparison of the structures of hDPP4 (PDB code: 8ZDX) and MjDPP4 (PDB code: 8ZE6). (Left panel) Crystal structure of hDPP4. (Middle panel) Crystal structure of MjDPP4. (Right panel) Superposition of hDPP4 and MjDPP4. hDPP4 and MjDPP4 are colored as in Fig 1B and 1C.
(TIF)

S4 Fig Comparison of the orientations of different β-coronaviruses RBDs with respect to DPP4. **(A)** Structural alignment of MjHKU4r-CoV-1 RBD–hDPP4 and MjHKU4r-CoV-1 RBD–MjDPP4 complexes, **(B)** MjHKU4r-CoV-1 RBD–MjDPP4 and Ty-BatCoV-HKU4 RBD-hDPP4 complexes, **(C)** MERS-CoV RBD–hDPP4 and Ty-BatCoV-HKU4 RBD-hDPP4 complexes. Shift distance and tilting angles between respective RBDs with DPP4 molecules aligned are labelled accordingly. **(D)** Superimposition of the four RBD-DPP4 complexes as shown in (A), (B) and (C). **(E)** Structural alignment of RaTG13 RBD-hACE2 (PDB codes: 7DRV) and SARS-CoV RBD-hACE2 (PDB codes: 2AJF) complexes. **(F)** Superimposition of the structures of human ACE2 complexed with RBDs from five SARS-CoV related coronaviruses, including SARS-CoV (PDB code: 2AJF), SARS-CoV-2 (PDB code: 6M0J), bat coronavirus RaTG13 (PDB code: 7DRV), SARS-CoV-2 Delta variant (PDB code: 7W9I), SARS-CoV-2 Omicron variant (PDB code: 7U0N). These viral RBDs are colored in cyan, blue, salmon, dark green and light green, respectively, whereas the human ACE2 molecules in these complexes are colored in yellow.
(TIF)

S5 Fig **The composite-omit maps contoured at 1.0σ for residues at the binding interfaces of MjHKU4r-CoV-1 RBD-hDPP4 and MjHKU4r-CoV-1 RBD-MjDPP4 complexes (A-D).** The MjHKU4r-CoV-1 RBD, hDPP4 and MjDPP4 are colored in light pink, wheat and pale cyan, respectively.
(TIF)

S6 Fig Interaction of wild-type (WT) or mutant MjHKU4r-CoV-1 RBD/Ty-BatCoV-HKU4 RBD with hDPP4/MjDPP4. **(A-B)** Binding of wild-type or mutant MjHKU4r-CoV-1 RBD (MjHKU4r-RBD) to hDPP4 or MjDPP4 measured by SPR. K_D_ values are expressed as the mean ± SEM (standard errors of the means), n ≥ 2. Kinetic model was used for analysis. The fitted curve is represented by dashed line. **(C)** Interaction network of MjHKU4r-CoV-1 RBD residues D471, Y506, Q508 with hDPP4 R336. 2Fo-Fc maps contoured at 1.0σ for these residues are shown as grey mesh. MjHKU4r-CoV-1 RBD D471 interacts with RBD Y506 via water-bridged hydrogen bond. The minimum distance between atoms of RBD D471 and hDPP4 R336 is 5.7 Å. **(D-E)** Analysis of the interaction between MjHKU4r-CoV-1 RBD and MjDPP4. **(D)** The key residues with important contribution to the binding of the complex in the wild-type and mutant systems. **(E)** Contact heat map between interfacial residues of MjHKUr-CoV-1 RBD and MjDPP4: WT (at left), Mut (at right). **(F)** Binding of wild-type (WT) or mutant MjHKU4r-CoV-1 RBD to TpDPP4 measured by SPR.
(TIF)

S7 Fig Binding of wild-type (WT) or mutant MjHKU4r-CoV-1 RBD/Ty-BatCoV-HKU4 RBD to hDPP4/MjDPP4 measured by SPR. KD values are expressed as the mean ± SEM (standard errors of the means), n ≥ 2. Kinetic model was used for analysis. The fitted curve is represented by dashed line.
(TIF)

S8 Fig Sequence and structural comparisons of the receptor-binding domains (RBDs) of different merbecoviruses. **(A)** Sequence alignment of the RBDs from MERS-CoV RBD (JX869059), MjHKU4r-CoV-1 RBD (UVJ46720.1), Ty-BatCoV-HKU4-1 RBD (ABN10848.1), Ty-BatCoV-HKU4-2 RBD (EF065506.1), and NeoCoV RBD (AGY29650.2). The residues on the receptor-binding motifs (RBM) and core structures of these viral RBDs are shaded in yellow and gray, respectively. The residues on the MERS-CoV RBD that bind to hDPP4 are marked with red pentagons. The RBMs from MjHKU4r-CoV-1, Ty-BatCoV-HKU4-1, Ty-BatCoV-HKU4-2, and NeoCoV share sequence identities of 45.83%, 43.75%, 43.75%, and 15.63% with MERS-CoV RBM, respectively. The core structures of the viral RBDs from MjHKU4r-CoV-1, Ty-BatCoV-HKU4-1, Ty-BatCoV-HKU4-2, and NeoCoV share sequence identities of 65.52%, 59.31%, 60%, and 37.93% with MESR-CoV core structure, respectively. **(B)** Structural alignment of the RBMs from MERS-CoV, MjHKU4r-CoV-1 and Ty-BatCoV-HKU4-1. **(C)** Structural alignment of the RBMs from MERS-CoV and NeoCoV.
(TIF)

S1 Table Data Collection and Refinement Statistics. (PDF)

S2 Table Hydrogen bonds and salt bridges at the MjHKU4r-CoV-1 RBD and hDPP4/MjDPP4 interface. (PDF)

S3 Table List of contact residues between MjHKU4r-CoV-1 RBD and hDPP4 / MjDPP4. (PDF)

S4 Table Components of the MM/GBSA binding free energy between MjHKU4r-CoV-1 RBD and MjDPP4 based on the last 10-ns in the wild-type and mutant systems (kcal/mol). (XLSX)

S5 Table SPR association rate constants (k_a_) and dissociation rate constants (k_d_) of MjHKU4r-CoV-1 RBD (wild-type and mutants) or Ty-BatCoV-HKU4 RBD binding to hDPP4. (XLSX)
