# Supplementary figures and images for: Structural basis for human DPP4 receptor recognition by a pangolin MERS-like coronavirus
Source: PLoS Pathog. 2024 Nov 8;20(11):e1012695. doi: 10.1371/journal.ppat.1012695 (PMC11578449; doi:10.1371/journal.ppat.1012695)

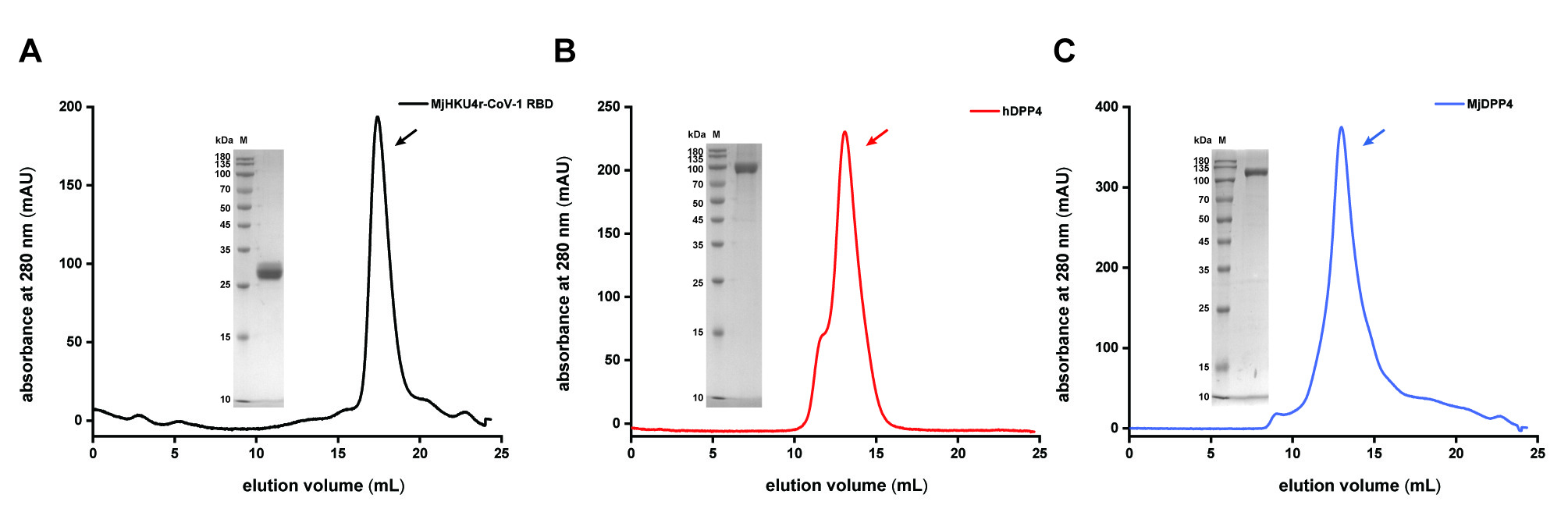

Supplement: S1 Fig — The size exclusion chromatograms of MjHKU4r-CoV-1 RBD (A), hDPP4 (B) and MjDPP4 (C). The proteins were purified by a Superdex 200 Increase 10/300 GL column (Cytiva). The pooled proteins, which are indicated by arrows, were further analyzed by SDS-PAGE. Lane 1, protein molecular weight marker. Lane 2, pooled proteins. (TIF) [file ppat.1012695.s001.tif]

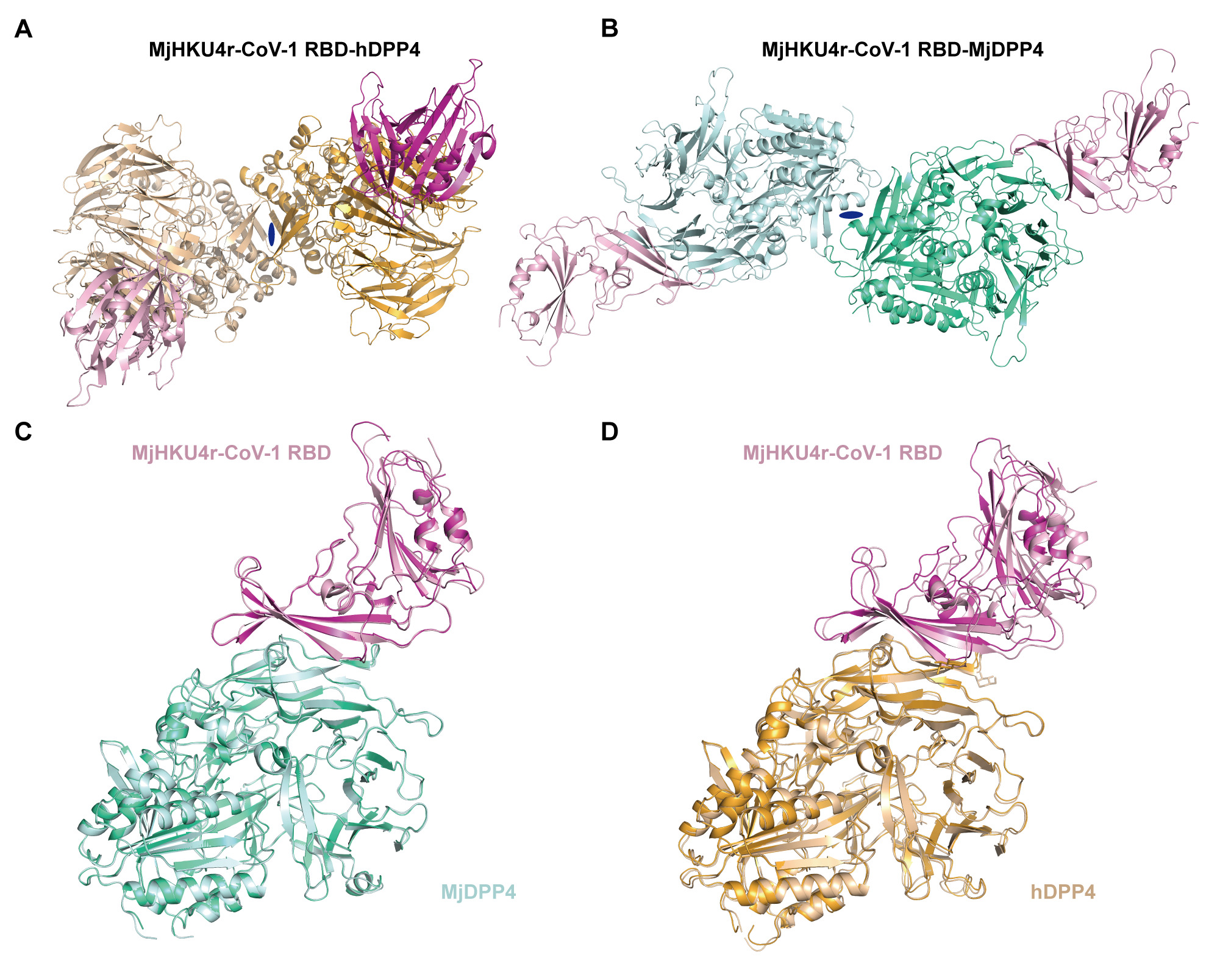

Supplement: S2 Fig — The two MjHKU4r-CoV-1 RBD-hDPP4 (A) and two MjHKU4r-CoV-1 RBD-MjDPP4 (B) heterocomplexes in the ASUs are related by non-crystallographic 2-fold axes (represented as lens-shaped symbols). (C) Structural alignments of the two MjHKU4r-CoV-1 RBD-MjDPP4 complexes in the ASU. (D) Superimposition of two MjHKU4r-CoV-1 RBD-hDPP4 complexes in the ASU. (TIF) [file ppat.1012695.s002.tif]

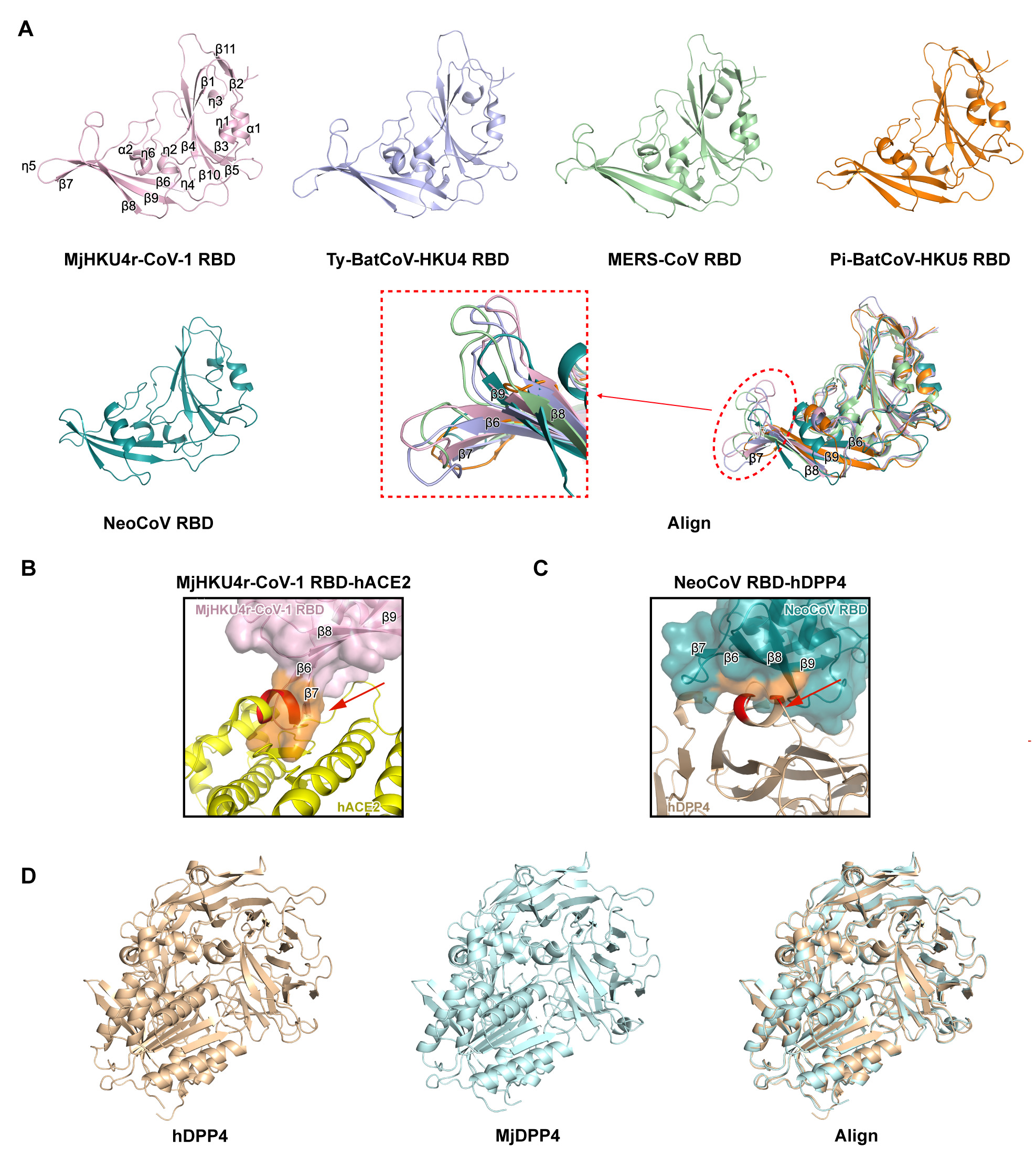

Supplement: S3 Fig — (A) Superimposition of the structures of MjHKU4r-CoV-1 RBD (PDB code: 8ZDX), Ty-BatCoV-HKU4 RBD (PDB code: 4QZV), MERS-CoV RBD (PDB code: 4KR0), Pi-BatCoV-HKU5-CoV RBD (PDB code: 5XGR) and NeoCoV RBD (PDB code: 7WPO). Zoom-in view of RBM from different merbecoviruses was shown. (B) Alignment of MjHKU4r-CoV-1 RBD to NeoCoV RBD-ACE2 complex (PDB code: 7WPO). The arrow indicates clashes between MjHKU4r-CoV-1 RBD and ACE2. (C) Alignment of NeoCoV RBD to MjHKU4r-CoV-1 RBD-hDPP4 complex. The arrow points to clashes between NeoCoV RBD and hDPP4. (D) Comparison of the structures of hDPP4 (PDB code: 8ZDX) and MjDPP4 (PDB code: 8ZE6). (Left panel) Crystal structure of hDPP4. (Middle panel) Crystal structure of MjDPP4. (Right panel) Superposition of hDPP4 and MjDPP4. hDPP4 and MjDPP4 are colored as in Fig 1B and 1C. (TIF) [file ppat.1012695.s003.tif]

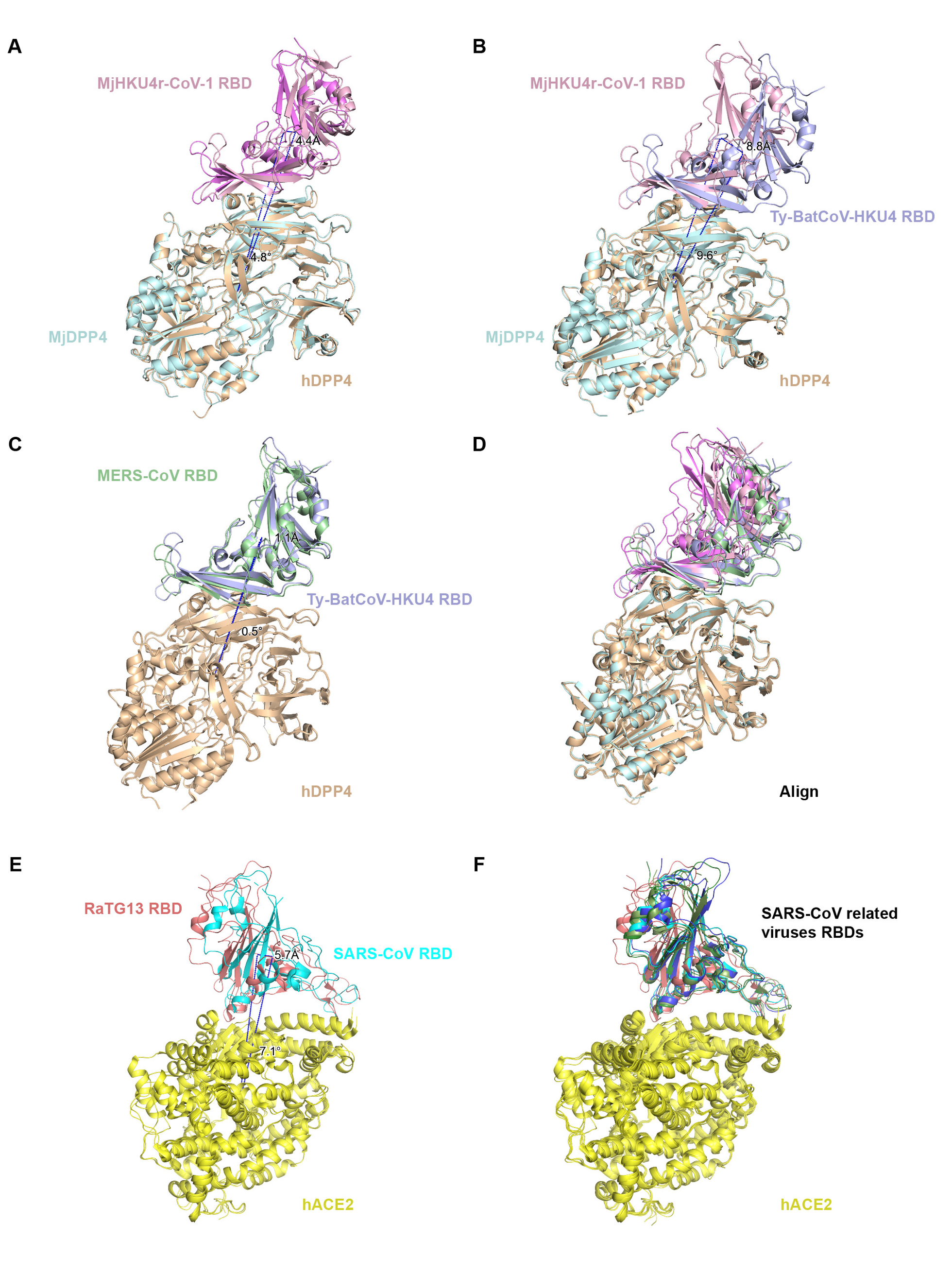

Supplement: S4 Fig — (A) Structural alignment of MjHKU4r-CoV-1 RBD–hDPP4 and MjHKU4r-CoV-1 RBD–MjDPP4 complexes, (B) MjHKU4r-CoV-1 RBD–MjDPP4 and Ty-BatCoV-HKU4 RBD-hDPP4 complexes, (C) MERS-CoV RBD–hDPP4 and Ty-BatCoV-HKU4 RBD-hDPP4 complexes. Shift distance and tilting angles between respective RBDs with DPP4 molecules aligned are labelled accordingly. (D) Superimposition of the four RBD-DPP4 complexes as shown in (A), (B) and (C). (E) Structural alignment of RaTG13 RBD-hACE2 (PDB codes: 7DRV) and SARS-CoV RBD-hACE2 (PDB codes: 2AJF) complexes. (F) Superimposition of the structures of human ACE2 complexed with RBDs from five SARS-CoV related coronaviruses, including SARS-CoV (PDB code: 2AJF), SARS-CoV-2 (PDB code: 6M0J), bat coronavirus RaTG13 (PDB code: 7DRV), SARS-CoV-2 Delta variant (PDB code: 7W9I), SARS-CoV-2 Omicron variant (PDB code: 7U0N). These viral RBDs are colored in cyan, blue, salmon, dark green and light green, respectively, whereas the human ACE2 molecules in these complexes are colored in yellow. (TIF) [file ppat.1012695.s004.tif]

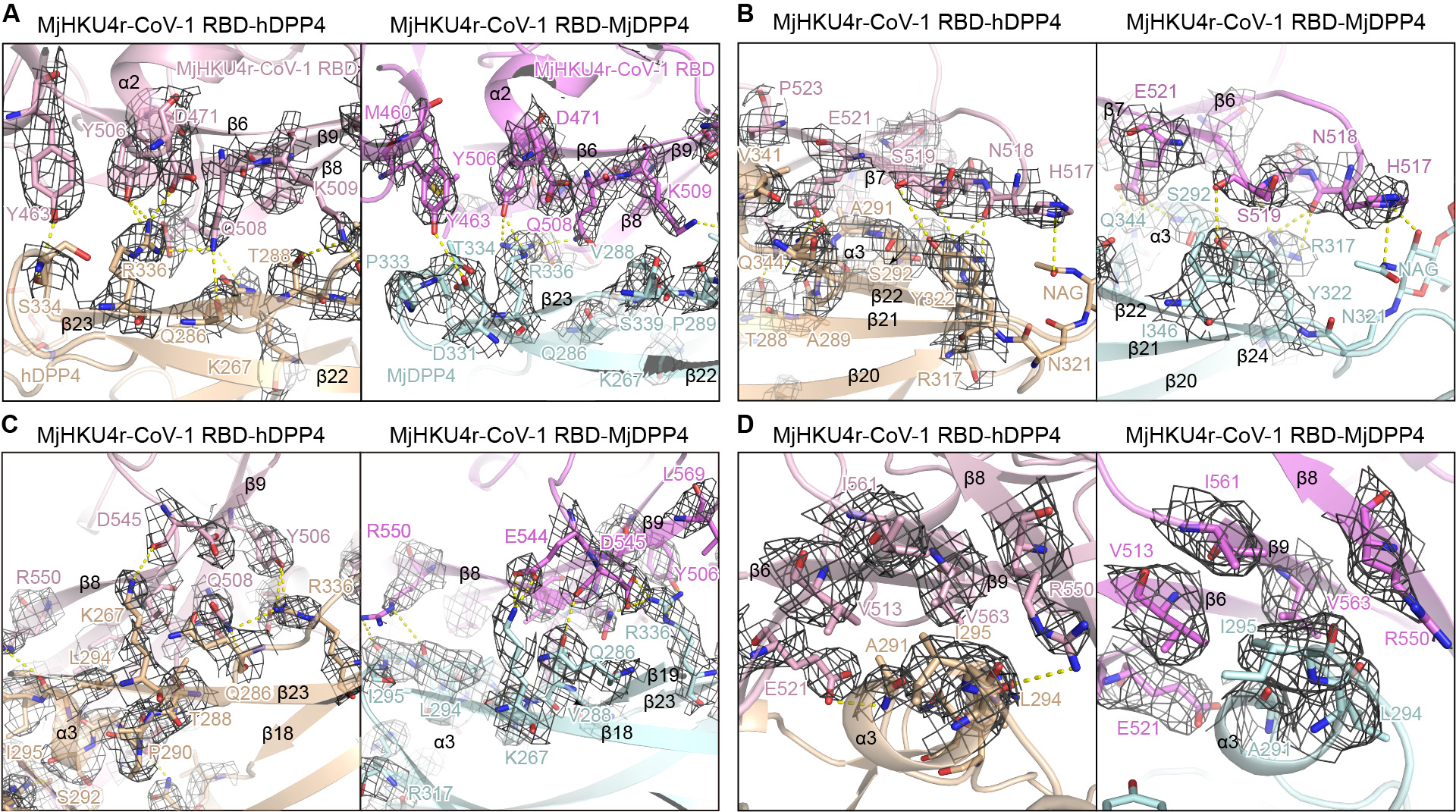

Supplement: S5 Fig — The composite-omit maps contoured at 1.0σ for residues at the binding interfaces of MjHKU4r-CoV-1 RBD-hDPP4 and MjHKU4r-CoV-1 RBD-MjDPP4 complexes (A-D). The MjHKU4r-CoV-1 RBD, hDPP4 and MjDPP4 are colored in light pink, wheat and pale cyan, respectively. (TIF) [file ppat.1012695.s005.tif]

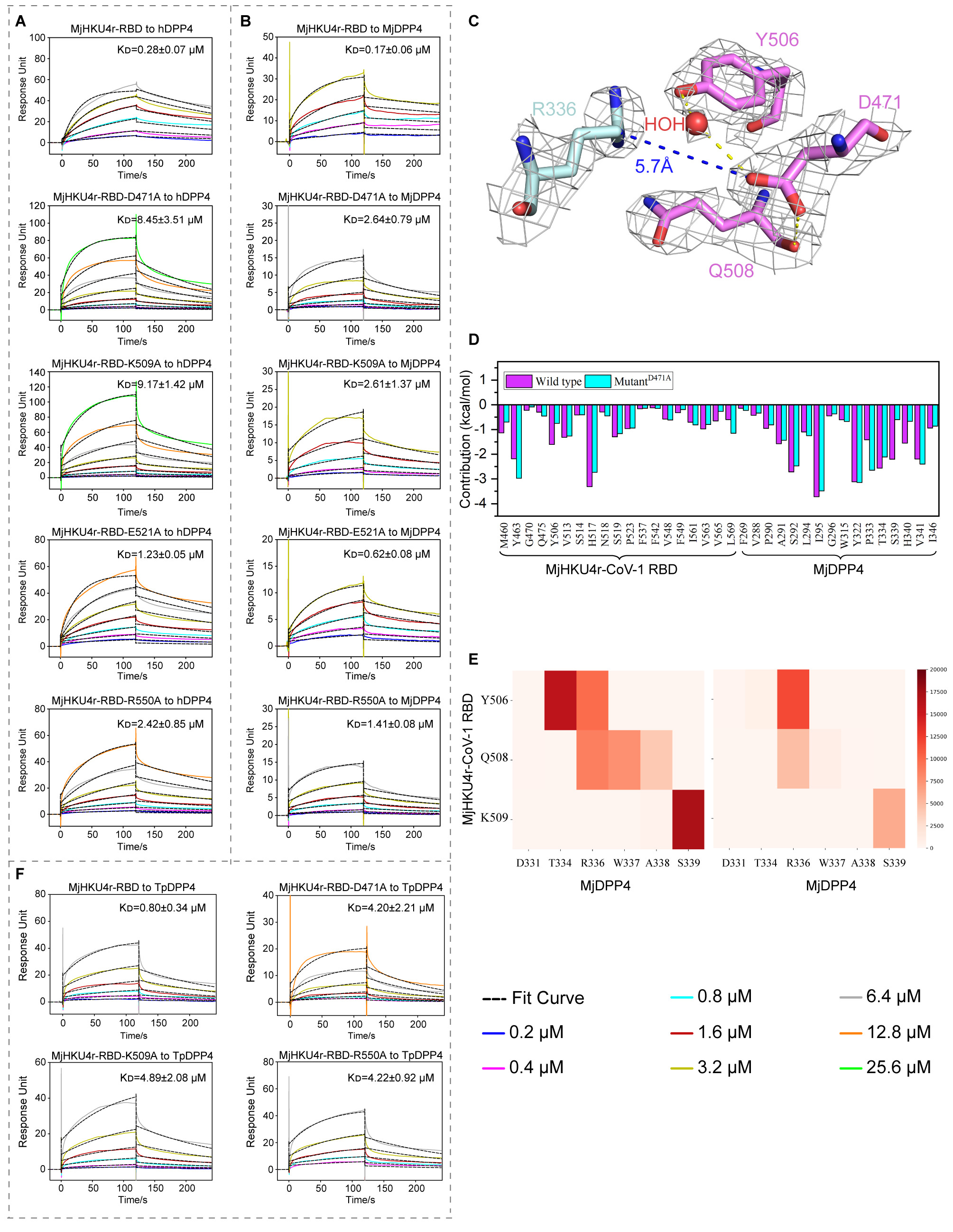

Supplement: S6 Fig — (A-B) Binding of wild-type or mutant MjHKU4r-CoV-1 RBD (MjHKU4r-RBD) to hDPP4 or MjDPP4 measured by SPR. KD values are expressed as the mean ± SEM (standard errors of the means), n ≥ 2. Kinetic model was used for analysis. The fitted curve is represented by dashed line. (C) Interaction network of MjHKU4r-CoV-1 RBD residues D471, Y506, Q508 with hDPP4 R336. 2Fo-Fc maps contoured at 1.0σ for these residues are shown as grey mesh. MjHKU4r-CoV-1 RBD D471 interacts with RBD Y506 via water-bridged hydrogen bond. The minimum distance between atoms of RBD D471 and hDPP4 R336 is 5.7 Å. (D-E) Analysis of the interaction between MjHKU4r-CoV-1 RBD and MjDPP4. (D) The key residues with important contribution to the binding of the complex in the wild-type and mutant systems. (E) Contact heat map between interfacial residues of MjHKUr-CoV-1 RBD and MjDPP4: WT (at left), Mut (at right). (F) Binding of wild-type (WT) or mutant MjHKU4r-CoV-1 RBD to TpDPP4 measured by SPR. (TIF) [file ppat.1012695.s006.tif]

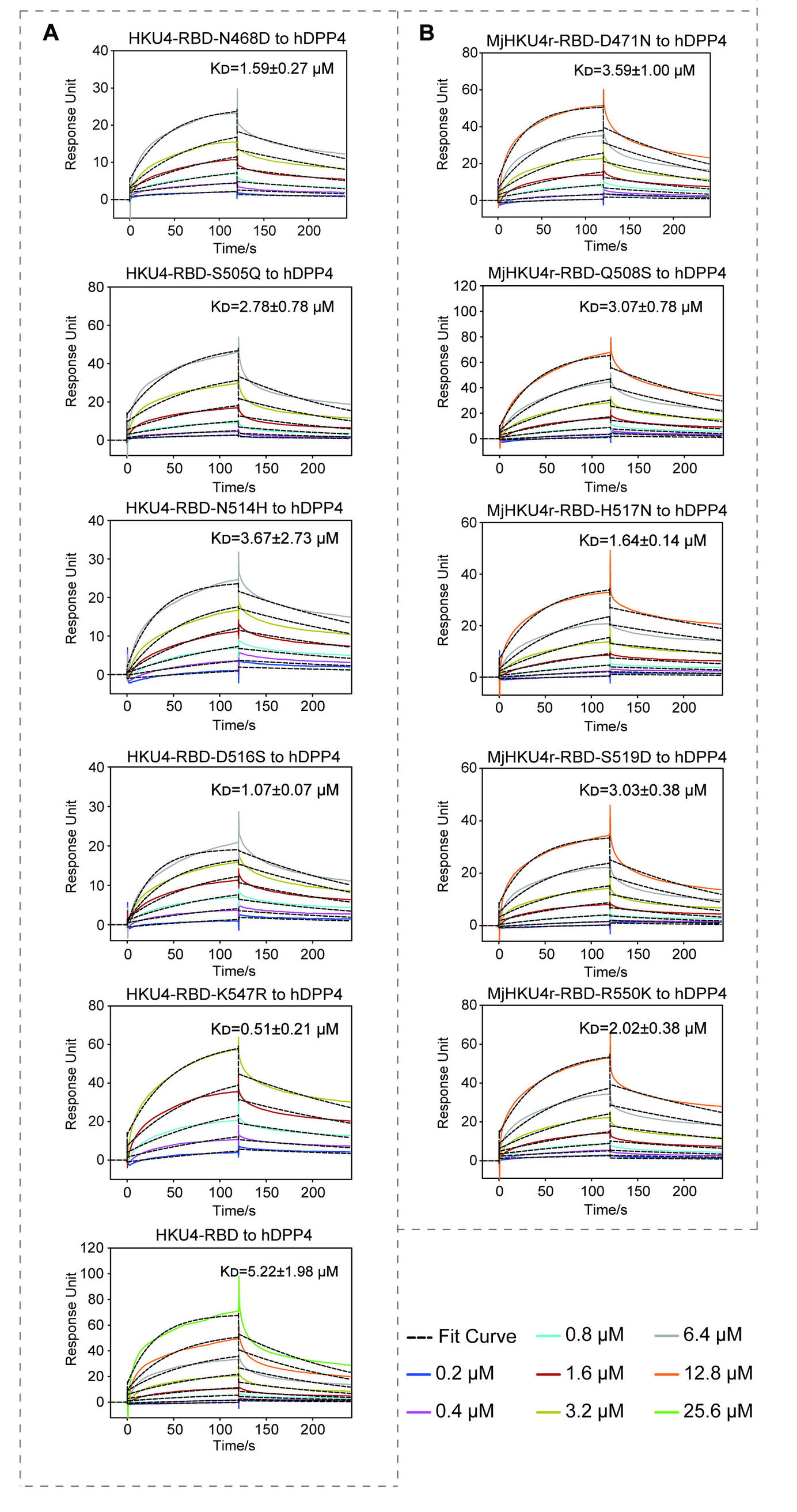

Supplement: S7 Fig — KD values are expressed as the mean ± SEM (standard errors of the means), n ≥ 2. Kinetic model was used for analysis. The fitted curve is represented by dashed line. (TIF) [file ppat.1012695.s007.tif]

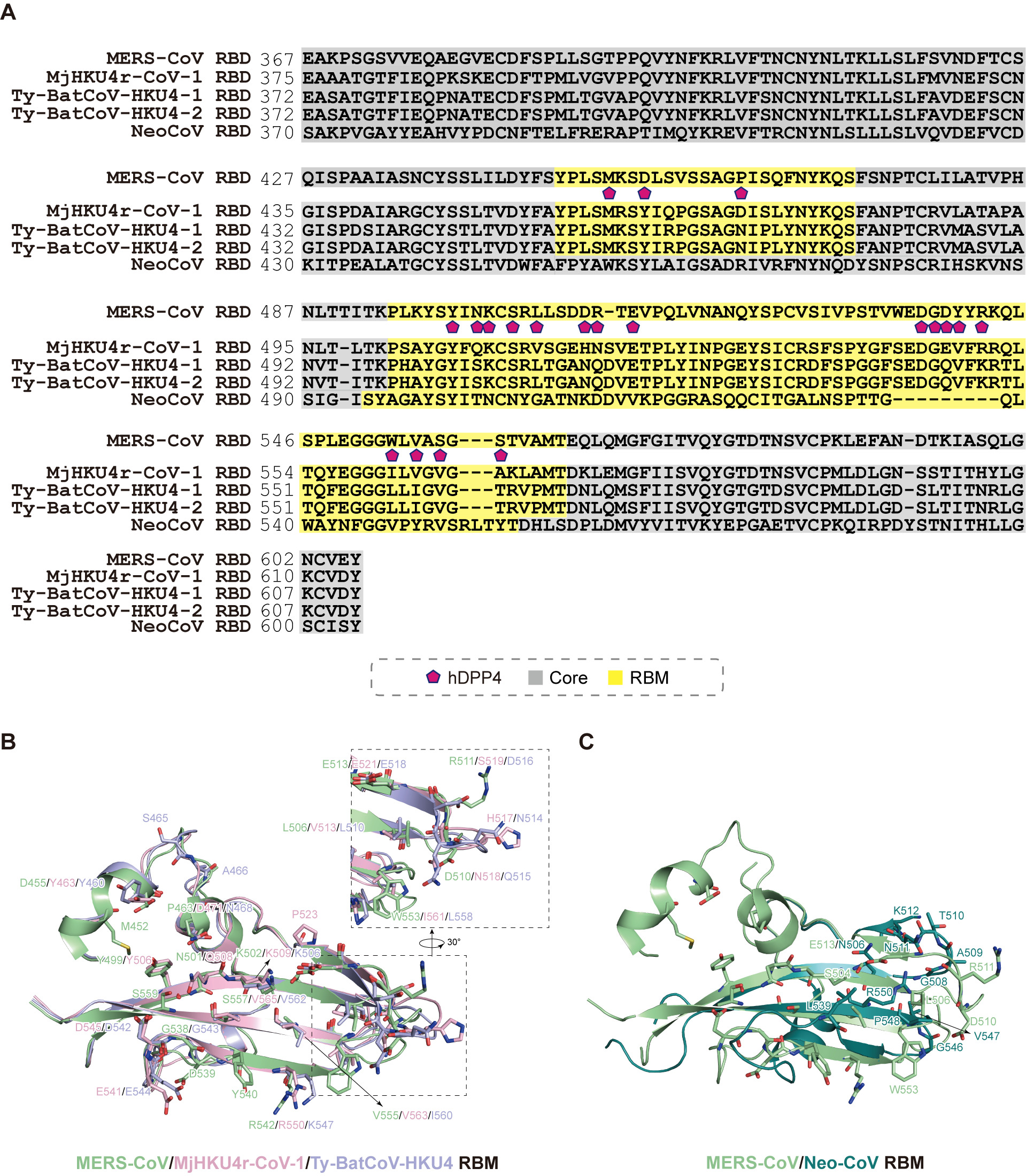

Supplement: S8 Fig — (A) Sequence alignment of the RBDs from MERS-CoV RBD (JX869059), MjHKU4r-CoV-1 RBD (UVJ46720.1), Ty-BatCoV-HKU4-1 RBD (ABN10848.1), Ty-BatCoV-HKU4-2 RBD (EF065506.1), and NeoCoV RBD (AGY29650.2). The residues on the receptor-binding motifs (RBM) and core structures of these viral RBDs are shaded in yellow and gray, respectively. The residues on the MERS-CoV RBD that bind to hDPP4 are marked with red pentagons. The RBMs from MjHKU4r-CoV-1, Ty-BatCoV-HKU4-1, Ty-BatCoV-HKU4-2, and NeoCoV share sequence identities of 45.83%, 43.75%, 43.75%, and 15.63% with MERS-CoV RBM, respectively. The core structures of the viral RBDs from MjHKU4r-CoV-1, Ty-BatCoV-HKU4-1, Ty-BatCoV-HKU4-2, and NeoCoV share sequence identities of 65.52%, 59.31%, 60%, and 37.93% with MESR-CoV core structure, respectively. (B) Structural alignment of the RBMs from MERS-CoV, MjHKU4r-CoV-1 and Ty-BatCoV-HKU4-1. (C) Structural alignment of the RBMs from MERS-CoV and NeoCoV. (TIF) [file ppat.1012695.s008.tif]
