# Supplementary material for: Structural basis for human DPP4 receptor recognition by a pangolin MERS-like coronavirus
Source: PLoS Pathog. 2024 Nov 8;20(11):e1012695. doi: 10.1371/journal.ppat.1012695 (PMC11578449; doi:10.1371/journal.ppat.1012695)
Supplement: S1 Table — (PDF) [file ppat.1012695.s009.pdf]

S1 Table. Data Collection and Refinement Statistics

	MjHKU4r-CoV-1-hDPP4 complex	MjHKU4r-CoV-1-MjDPP4 complex
**Data collection**		
Space group	P21	P21
Cell dimensions		
a, *b*, *c* (Å)	74.98, 121.90, 144.66	96.53, 121.44, 110.62
α, β, γ (°)	90.00, 93.82, 90.00	90.00, 105.13, 90.00
Resolution (Å)[a]	20.00-2.60 (2.75-2.60)	50.00-2.70 (2.87-2.70)
Unique reflections	79444(12098)	66775 (10670)
R_{merge}[a, b]	0.167 (3.079)	0.315 (2.399)
R_{pim}	0.068 (1.368)	0.183 (1.507)
CC1/2	0.997 (0.372)	0.971 (0.372)
$I / \sigma(I)$[a]	8.85 (0.55)	4.98(0.76)
Completeness (%)[a]	99.0 (95.3)	98.8 (98.4)
Redundancy[a]	6.95 (7.06)	3.88 (3.92)
**Refinement**		
Resolution (Å)	19.69-2.60	47.49-2.70
No. reflections	78995	66672
R_{work} / R_{free}[c]	0.21/0.25	0.24/0.27
No. atoms		
Protein	15152	15030
Ligand/Ion	261	244
Water	23	157
B-factors ($Å^2$)		
Protein	98.53	66.27
Ligand/Ion	121.32	77.08
Water	68.41	51.99
R.m.s. deviations		
Bond lengths (Å)	0.002	0.002
Bond angles (°)	0.497	0.491
Ramachandran plot(%)[d]		
Favored	95.06	94.64
Allowed	4.83	5.20
Outliers	0.11	0.16

[a]Values for the outmost resolution shell are given in parentheses.

[b]$R_{merge}=\Sigma_i\Sigma_{hkl}| I_i - \langle I\rangle | / \Sigma_i\Sigma_{hkl} I_i$, where I_i is the observed intensity and $\langle I\rangle$ is the average intensity from multiple measurements.

[c]$R_{work} = \Sigma | | F_o| - | F_c| | / \Sigma | F_o|$, where F_o and F_c are the structure-factor amplitudes from the data and the model, respectively. R_{free} is the R factor for a subset (5%) of reflections that was selected prior to refinement calculations and was not included in the refinement.

[d]Ramachandran plots were generated by using the program MolProbity.
