# Supplementary material for: Structural basis for human DPP4 receptor recognition by a pangolin MERS-like coronavirus
Source: PLoS Pathog. 2024 Nov 8;20(11):e1012695. doi: 10.1371/journal.ppat.1012695 (PMC11578449; doi:10.1371/journal.ppat.1012695)
Supplement: S2 Table — (PDF) [file ppat.1012695.s010.pdf]

S2 Table. Hydrogen bonds and salt bridges at the MjHKU4r-CoV-1 RBD and hDPP4/MjDPP4 interface

	hDPP4	Length(Å)	MjHKU4r-CoV-1 RBD	Length(Å)	MjDPP4
			Y463(OH)	3.97	D331(OD1)
**Hydrogen bonds**	S334(O)	3.64	Y463(OH)	3.62	D331(OD2)
	R336(NH1)	2.91	Y506(OH)	3.00	R336(NH1)
	R336(NH2)	3.87	Y506(OH)	3.33	R336(NE)
	Q286(O)	3.58	Q508(NE2)	3.21	R336(NH2)
	R336(NH2)	3.32	Q508(NE2)		
	T288(OG1)	3.15	K509(NZ)	3.31	A291(N)
	A289(O)	2.92	K509(NZ)		
	A291(N)	3.90	K509(NZ)		
	R317(NH1)	3.07	H517(O)	3.72	R317(NH1)
	R317(NH2)	3.01	H517(O)	3.44	R317(NH2)
	809NAG	3.85	H517(O)	2.73	808NAG
			H517(O)	3.69	808NAG
	R317(NH2)	2.95	N518(O)	2.77	R317(NH2)
	Y322(OH)	3.90	S519(O)	3.50	Y322(OH)
	Q344(NE2)	3.00	E521(OE1)	2.98	A291(N)
	Q344(OE1)	3.54	E521(OE1)	3.66	S292(N)
	A291(N)	3.66	E521(OE2)	3.41	A291(N)
	A291(N)	3.19	E521(OE1)	3.55	A291(N)
			E521(OE1)	3.75	Q344(OE1)
			Y525(OH)	2.35	R342(NH2)
	K267(NZ)	3.59	D545(O)	3.16	Q286(OE1)
			R550(NH1)	3.41	I295(O)
	L294(O)	3.36	R550(NH2)		
	L294(O)	3.20	R550(NE)	3.90	L294(O)
	I295(O)	3.28	R550(NE)		
**Salt bridges**	R336(NH1)	2.53	D471(OD2)		
			E544(OE1)	2.76	K267(NZ)
			E544(OE2)	3.58	K267(NZ)
			D545(OD1)	2.65	R336(NH2)
			D545(OD2)	3.06	R336(NH2)

ND2, nitrogen delta 2; NE2, nitrogen epsilon 2; NZ, nitrogen zeta; N, nitrogen; NH1, nitrogen eta 1; NH2, nitrogen eta 2; OH, oxygen eta; O, oxygen; OD1, oxygen delta 1; OD2, oxygen delta 2; OG1, oxygen gamma 1; OE1, oxygen epsilon 1; OE2, oxygen epsilon 2.
