# Supplementary material for: Structural basis for human DPP4 receptor recognition by a pangolin MERS-like coronavirus
Source: PLoS Pathog. 2024 Nov 8;20(11):e1012695. doi: 10.1371/journal.ppat.1012695 (PMC11578449; doi:10.1371/journal.ppat.1012695)
Supplement: S3 Table — (PDF) [file ppat.1012695.s011.pdf]

S3 Table. List of contact residues between MjHKU4r-CoV-1 RBD and hDPP4 / MjDPP4

MjHKU4r-CoV-1 RBD	hDPP4	MjDPP4
**M460**		T334
Y463	S334	D331 P333 T334
**D471**	R336	
Y506	R336	R336
Q508	Q286 R336	V288 R336
K509	T288 A289 P290 A291	V288 P289 P290 A291 S339
**S511**		A291
V513	A291	A291 S292 I295
H517	R317 809NAG	R317 808NAG 809NAG
N518	S292 R317	I295 R317
S519	Y322	Y322 I346
E521	P290 A291 V341 Q344	A291 S292 V341 Q344
**P523**	V341	
**Y525**		R342
E544	NAG 806	K267
D545	K267	Q286 R336
R550	L294 I295	L294 I295 G296
I561	I295	I295
V563	A291 L294 I295	A291 I295
V565	L294	V288 L294
**A567**		R336
**L569**		R336

A distance cut-off of 4 Å was used. MjHKUr-CoV-1 RBD residues in bold are involved in interactions that are not shared by the two DPP4 molecules.
